# Temporal Stability and the Effect of Transgenerational Transfer on Fecal Microbiota Structure in a Long Distance Migratory Bird

**DOI:** 10.3389/fmicb.2017.00050

**Published:** 2017-02-01

**Authors:** Jakub Kreisinger, Lucie Kropáčková, Adéla Petrželková, Marie Adámková, Oldřich Tomášek, Jean-François Martin, Romana Michálková, Tomáš Albrecht

**Affiliations:** ^1^Department of Zoology, Faculty of Science, Charles UniversityPrague, Czechia; ^2^Department of Ecology, Faculty of Science, Charles UniversityPrague, Czechia; ^3^Institute of Vertebrate Biology, Czech Academy of Sciences, StudenecCzechia; ^4^Montpellier-SupAgro, UMR Centre de Biologie pour la Gestion des PopulationsMontferrier-sur-Lez, France

**Keywords:** microbiome, metagenome, symbiosis, gastrointestinal tract, barn swallow, fecal microbiota

## Abstract

Animal bodies are inhabited by a taxonomically and functionally diverse community of symbiotic and commensal microorganisms. From an ecological and evolutionary perspective, inter-individual variation in host-associated microbiota contributes to physiological and immune system variation. As such, host-associated microbiota may be considered an integral part of the host’s phenotype, serving as a substrate for natural selection. This assumes that host-associated microbiota exhibits high temporal stability, however, and that its composition is shaped by trans-generational transfer or heritable host-associated microbiota modulators encoded by the host genome. Although this concept is widely accepted, its crucial assumptions have rarely been tested in wild vertebrate populations. We performed 16S rRNA metabarcoding on an extensive set of fecal microbiota (FM) samples from an insectivorous, long-distance migratory bird, the barn swallow (*Hirundo rustica*). Our data revealed clear differences in FM among juveniles and adults as regards taxonomic and functional composition, diversity and co-occurrence network complexity. Multiple FM samples from the same juvenile or adult collected within single breeding seasons exhibited higher similarity than expected by chance, as did adult FM samples over two consecutive years. Despite low effect sizes for FM stability over time at the community level, we identified an adult FM subset with relative abundances exhibiting significant temporal consistency, possibly inducing long-term effects on the host phenotype. Our data also indicate a slight maternal (but not paternal) effect on FM composition in social offspring, though this is unlikely to persist into adulthood. We discuss our findings in the context of both evolution and ecology of microbiota vs. host interactions and barn swallow biology.

## Introduction

The bodies of animals are inhabited by taxonomically and functionally diverse communities of symbiotic and commensal microorganisms (Qin et al., 2010; Muegge et al., 2011). Recent advances in this field have clearly shown that such host-associated microbiota provide important benefits to the host. In particular, microbiota modulate development of digestive tract morphology (Reikvam et al., 2011), enable synthesis of essential bioactive molecules that cannot be synthesized by the host (Bäckhed et al., 2005), stimulate the host’s immune system (Macpherson and Harris, 2004; Wu and Wu, 2012) and provide protection against pathogens (Koch and Schmid-Hempel, 2011). In addition, unlike host-encoded enzymes, enzymes encoded by the microbial metagenome enable cleavage of complex substrates such as plant polysaccharides. Products of these pathways can be further processed by the host’s metabolism and, consequently, microbial communities positively contribute to the host’s energy balance (Jumpertz et al., 2011). In addition to these benefits, however, certain species of host-associated microbiota can induce harmful effects, such as reduced diet processing capacity (Smith et al., 2013), chronic inflammation (Dapito et al., 2012), or production of toxins (Yoshimoto et al., 2013).

In vertebrates, host-associated microbiota typically exhibit pronounced variation at the inter-individual level (Baxter et al., 2015; Kreisinger et al., 2015b; Yuan et al., 2015; Lewis et al., 2016). Despite a degree of functional redundancy as regards genes encoded by individual host-associated microbiota taxa (Moya and Ferrer, 2016), such variation underlines inter-individual differences in health status and a wide range of physiological and body-condition traits (Macpherson and Harris, 2004; Koch and Schmid-Hempel, 2011; Smith et al., 2013). With respect to this inter-individual variation a number of obvious questions arise, one of the most important is to what extent does individual specificity in host-associated microbiota composition vary over time.

Hosts may exhibit a certain degree of tolerance to newly invading bacterial species; at the same time, the abundance of microbiota already present may change due to switches in host diet and physiological state (Jumpertz et al., 2011; David et al., 2014; Salonen et al., 2014; Wang et al., 2014). Such changes in microbiota over time may increase the host’s ability to cope with new environmental challenges. Under certain circumstances, however, such changes in microbiota composition could have no effect or induce adverse effects on the host (DiBaise et al., 2012; Kumar et al., 2016). As a result, multicellular organisms have evolved a plethora of mechanisms aimed at maintenance of beneficial microbial taxa and suppression of potentially detrimental microbes (Salminen et al., 2004; Janson et al., 2008; Benson et al., 2010; McKnite et al., 2012). Probably the best known of these host-intrinsic factors are those genes predominately involved in regulation of the immune system, which have a long-standing effect on associate microbial communities (Benson et al., 2010; McKnite et al., 2012; Bolnick et al., 2014; Wang et al., 2015). Given that immune genes typically display high allelic variation (Sommer, 2005), they could contribute to both long-term stability and inter-individual variation of host-associate microbiota. In addition to genetic factors, trans-generational transfer of microbiota could have a long-standing effect on microbiota composition in the progeny of parental generations (Salminen et al., 2004). In some taxa, this may even result in co-divergence between microbiota and host phylogenies over their evolutionary history (Sanders et al., 2014). Both trans-generational transfer and genetic regulation of microbial communities imply some level of heritability in host-associated microbiota. Consequently, if host-associated microbiota exhibit both long-term stability and heritability, this could be regarded as an extension of host-heritable phenotype variation and serve as the substrate for natural selection (Zilber-Rosenberg and Rosenberg, 2008; Bordenstein and Theis, 2015).

Current evidence for the temporal stability and heritability of host-associated microbiota is still rather puzzling, however, and has been addressed by a surprisingly low number of studies. Furthermore, what studies there have been have tended to focus mainly on human populations and captive-bred model species (Benskin et al., 2010; Schloss et al., 2012; Faith et al., 2013; Lim et al., 2014; Salonen et al., 2014; Tap et al., 2015). Limited effort has been aimed at assessing the strength of temporal stability and mechanisms affecting host-associated microbiota establishment during ontogeny in wild populations (Waite et al., 2014; Baxter et al., 2015; Sun et al., 2016). Great care should be taken when extrapolating results obtained in captivity or from humans to microbiota vs. host interactions in wild populations. In the case of human populations, long-term host-associated microbiota stability could be affected to a large degree by long-term stability of life-style, including diet preferences, exposure to stressors modulating host-associated microbiota and other traits associated with micro-culture variation. In the case of laboratory-reared animals, the composition and functional properties of host-associated microbiota are typically distinct compared to wild populations (Xenoulis et al., 2010; Amato, 2013; Kreisinger et al., 2014). Consequently, captivity may induce large effects on the shape of interactions between host-associated microbiota and host physiology (Kreisinger et al., 2015a). In addition, environmental factors contributing to host-associated microbiota variation over time, such as variation in diet composition and environmental stressors, are typically homogeneous among individuals in breeding facilities. As a consequence, factors contributing to individual host-associated microbiota stability over time, such host genes interacting with host-associated microbiota members and/or vertical transfer of host-associated microbiota from parents to progeny, may be of higher importance in breeding facilities compared to wild populations. Last, but not least, most of our current knowledge on host-associated microbiota vs. host interaction relies on studies performed on mammals. Such taxonomic bias may affect our general view of the ecological and evolutionary factors associated with host-associated microbiota vs. host interaction as gut morphology and factors contributing to host-associated microbiota establishment and host-associated microbiota composition exhibit considerable taxon specificity in mammals. Unlike most other vertebrate taxa, mammals are typically viviparous. Physical contact between newborn young and the female’s vaginal microbiota during the delivery is important for host-associated microbiota colonization and this type of transfer has a long-term effect on microbiota composition (Salminen et al., 2004).

Passerines are an important model group for evolutionary, eco-physiological, and eco-immunological research (Bennett and Owens, 2002), particularly as we have a detailed knowledge on their physiology. However, the role of microbiota is still understudied in this group. In addition, passerines have a clearly distinct composition of host-associated microbial communities compared, to the more widely studied mammals (Hird et al., 2015; Kreisinger et al., 2015b; Lewis et al., 2016). This makes them an interesting and complementary model group for research on the ecological and evolutionary consequences of host vs. microbiota interaction. To date, the microbial community of the lower intestine, with microbiota in fecal samples typically used as a proxy, has been the most widely studied subset of animal-associated microbiota (Ley et al., 2008; Baxter et al., 2015; Hird et al., 2015; Lewis et al., 2016). Fecal microbiota (FM) differences between passerines and mammals are putatively associated with differences in gut anatomy and function. In particular, overall gut length and food retention time tends to be shorter in birds compared with mammals of similar body size (Caviedes-Vidal et al., 2007; McWhorter et al., 2009). Furthermore, those gut sections involved in bacterial fermentation, i.e., the caecum and colon, are typically reduced in passerines (Ruiz-Rodríguez et al., 2009). Unlike mammals, passerine FM may also be affected by direct contact with uric acid as the urine is conveyed to the cloaca directly by the kidney ureters (Braun and Campbell, 1989) and a number of bacterial species are capable of using uric acid as a substrate (Potrikus and Breznak, 1981; Thong-On et al., 2012). As in the case of mammals, however, microbiota may also be transferred from parents to progeny during physical contact in the course of food provisioning (Lucas and Heeb, 2005). However, little is known about the temporal stability of FM in passerines, or about the FM development during post-hatching ontogeny (Benskin et al., 2010; González-Braojos et al., 2012b).

In this contribution, we focus on temporal stability of FM in a passerine bird, the barn swallow (*Hirundo rustica*). The barn swallow is a long-distance migrant nesting in colonies with a complex social system (Petrželková et al., 2015). It has been suggested that social interaction between colony members affects the structure of host-associated microbiota in this species (Kreisinger et al., 2015b). As a species, the barn swallow forages exclusively on diverse groups of flying insects (Turner, 1980). Our study benefits from this tight specialization as FM variation due to differences in diet are likely to be relatively low compared to omnivorous taxa (Ley et al., 2008; David et al., 2014). Despite extensive knowledge on barn swallow biology (Møller, 1994), there has been just one culture-independent study aimed at characterization of microbial communities in this species based on high-throughput sequencing (Kreisinger et al., 2015b).

Here, we apply extensive repeat FM sampling of adults and juveniles from three breeding barn swallow populations. Metataxonomic approach based on high-throughput sequencing of 16S rRNA amplicones and imputation of FM functional content via PICRUSt (Langille et al., 2013) were used to get an insight into developmental trajectories of FM taxonomic and functional composition in this species. Importantly, our data set allows us to estimate the level stability in individual FM composition over time. In addition, we tested if the level of temporal stability differs among adults and juveniles and if the temporal stability is driven by abundance invariance of FM members or by invariance of their presence vs. absence. Finally, we use this dataset to assess whether social contact between parents and offspring shapes FM composition.

## Materials and Methods

### Field Sampling and Parentage Assignment

Field sampling was conducted during the barn swallow breeding season (from May to August) on three populations [Šaloun farm, Lomnice nad Lužnicí (49°4’7.762”N, 14°42’36.521”E), Hamr farm, Lužnice (49°3’25.288”N, 14°46’10.82”E), and Obora (48°59’06.8”N, 14°46’48.5”E)] in the Třeboňsko Protected Landscape Area (Czech Republic, average distance between populations = 7.9 km). Adult FM was sampled during 2013 and 2014, whereas juvenile FM was only sampled during 2014, at 6, 9, and 12 days after hatching (hereafter “age-classes”). See Petrželková et al. (2015) for more details on field procedures. To collect fecal samples, adults were placed in a paper bag and young in a plastic beaker filled with paper towels, where they were kept for approx. 30 min. Feces were harvested using a sterile microbiological swab (Copan, Italy), placed in sterile DNA/RNA free cryotubes (Simport, Canada) and stored in liquid nitrogen or at -80°C for further laboratory analysis.

Individuals in each population had their polymorphic microsatellites genotyped, as described in Petrželková et al. (2015). These data, together with direct observations of individually marked adults, allowed us to determine the social parents for individual clutches and the presence of extra-pair (i.e., sired by a non-social male) or parasitic (i.e., laid by a non-social female) young within individual clutches, as described elsewhere (Petrželková et al., 2015).

We analyzed 448 barn swallow FM samples in total; 197 samples from juveniles (*n* = 99 individuals, mean no. samples per individual = 2.01) and 251 from adults (*n* = 131 individuals, mean no. samples per individual = 1.91; see **Supplementary Table S1**). Our data set also included FM samples from 31 social mother vs. offspring pairs and from 37 social father vs. offspring pairs sampled during 2014.

All field procedures were conducted in accordance with the Guidelines for Animal Care and Treatment of the European Union, and approved by the Animal Care and Use Committees at the Czech Academy of Sciences (041/2011), and Charles University in Prague (4789/2008-0).

### Microbiota Genotyping

Metagenomic DNA from fecal samples was extracted in a laminar flow cabinet using the PowerSoil DNA isolation kit (MO BIO Laboratories Inc., USA). To optimize the efficiency of DNA isolation, samples were homogenized using a MagnaLyzer (Roche, Switzerland) for 30 s at 6000 rpm and the DNA extracted was eluted to 50 μl of elution buffer. Following the recommendations of Klindworth et al. (2013), primers covering the V3-V4 variable region on bacterial 16S rRNA [i.e., S-D-Bact-0341-b-S-17 (CCTACGGGNGGCWGCAG) and S-D-Bact-0785-a-A-21 (GACTACHVGGGTATCTAATCC)] were used during the polymerase chain reaction (PCR) step. Both forward and reverse primers were tagged with 10 bp barcodes designed by TagGD software (Costea et al., 2013). For the PCR, we used 8 μl of KAPA HIFI Hot Start Ready Mix (Kapa Biosystems, USA), 0.37 μM of each primer and 7 μl of DNA template. PCR conditions were as follows: initial denaturation at 95°C for 5 min followed by 35 cycles each of 98°C (20 s), 61°C (15 s), and 72°C (40 s), and a final extension at 72°C (5 min). The PCR product, together with negative controls (PCR products for blank DNA isolates), were run on 1.5% agarose gel and the concentration of PCR product assessed based on gel band intensity using GenoSoft software (VWR International, Belgium). Samples were subsequently pooled at equimolar concentration, the pooled samples then being run on 1.5% agarose gel, with bands of appropriate size excised from the gel and purified using the High Pure PCR product Purification Kit (Roche, Switzerland) according to the manufacturer’s instructions. Sequencing adaptors were ligated using TruSeq nano DNA library preparation kits (Illumina, USA) and the resulting amplicon libraries sequenced on a single Miseq run (Illumina, USA) using v3 chemistry and 2 × 300 bp paired-end reads. We then prepared technical PCR duplicates for individual DNA samples. As there was high consistency in both FM composition (Procrustes correlation: *r* = 0.98, *p* < 0.0001) and FM diversity (Pearson’s *r* = 0.97, *p* < 0.0001) among technical replicates, we merged the sequences corresponding to individual samples for downstream analysis.

### Bioinformatic Processing of 16S rRNA Data

Pair-end Illumina reads were merged using PEAR (Zhang et al., 2014) and de-mutiplexed using mothur (Schloss et al., 2009) and custom R/Bioconductor scripts (available from the authors on request). We then used Lotus pipeline (Hildebrand et al., 2014) for quality filtering of FASTQ files. Sequences were excluded if the average quality score was lower than 30 or if the average quality score within a 50 bp sliding window decreased below 25. UCHIME (implemented in the Lotus pipeline; Edgar et al., 2011) was used alongside the gold.fna database^1^ for detection and elimination of chimeric sequences. The resulting 16S rRNA sequences were clustered at a 97% similarity threshold using UPARSE (Edgar, 2013) in order to define operational taxonomic units (OTUs). Taxonomic assignation of representative sequences for each OTU was performed using RDP classifier (Wang et al., 2007) and the GreenGenes reference database, version gg_13_5 (DeSantis et al., 2006). Representative sequences were further aligned using PyNAST (Caporaso et al., 2010a), the maximum likelihood tree being constructed using FastTree (Price et al., 2009). We considered OTUs assigned as “Chloroplast” (6.2% of read after quality filtering), or those not assigned to any bacterial phylum (0.6% of read after quality filtering), as diet contaminants or sequencing artifacts, respectively, and excluded them from all downstream analyses. The resulting OTU tables, sample metadata, OTU tree and taxonomic annotations for individual OTUs were merged into a phyloseq object (McMurdie and Holmes, 2013) for statistical analysis in R version 3.2.3 (R Core Team, 2015).

### Metagenomic Predictions

Functional composition of the FM was inferred based on predictive models integrated into the PICRUSt pipeline (Langille et al., 2013). In brief, this approach utilizes 16s rRNA reads and ancestral state reconstruction algorithms to predict the functional content of FM samples based on the gene content of known bacterial genomes. First, we mapped our high-quality sequences against GreenGenes reference OTUs (DeSantis et al., 2006) using 91, 93, 95, and 97% similarity thresholds and the closed reference algorithm implemented in QIIME (Caporaso et al., 2010b). Next, metagenomes were predicted using the default PICRUSt setup and classified according to the Kyoto Encyclopedia of Genes and Genomes (KEGG; Kanehisa and Goto, 2000). The resulting table, along with the predicted abundance of KEGG categories in individual samples, was used for downstream analysis.

The proportion of sequences unassigned to a reference OTU in GreenGenes (i.e., unusable for metagenomic prediction) was relatively high at the 97% similarity threshold (24.3%) and relatively low at the 95–91% similarity thresholds (range = 7.9–1.3%). Hence, we decided to use 95% similarity mapping for PICRUSt predictions in order to avoid potential bias associated with a high proportion of unmappable reads. Althougth 95% similarity mapping may compromise PICRUSt precission, the “mean nearest sequenced taxon index” (NSTI), i.e., the average branch length separating OTUs from a reference bacterial genome, was only 0.065. This value is lower than the NTSI for the microbiomes of most non-model species (Langille et al., 2013).

### Statistical Analysis

Using phyloseq (McMurdie and Holmes, 2013), we calculated sample-specific alpha diversity indices (number of OTUs observed, Chao1, Shannon index) and, using linear mixed effect models (LME) contained in the R package lme4 (Bates et al., 2015), tested whether there was any difference in alpha diversity among juveniles and adults and whether FM diversity varied among different juvenile age-classes. Individual identity was considered as a random effect and the effect of breeding colony was included as a covariate. The significance of the main effects was assessed based on deviance ratio tests.

Community-wide divergence in OTU and predicted KEGG composition was assessed using multivariate techniques based on community dissimilarity among samples. Four ecological dissimilarity types were applied, each capturing different aspects of FM divergence: weighted and unweighted UniFrac (Lozupone and Knight, 2005), Bray–Curtis and a binary version of Jaccard dissimilarity. Jaccard and unweighted UniFrac dissimilarity only account for OTU presence vs. absence; hence, they are more sensitive than Bray–Curtis and weighted UniFrac dissimilarity to FM changes driven by rare OTUs. In addition, both unweighted and weighted UniFrac dissimilarity take account of OTU genetic similarity and, therefore, are more sensitive to community divergence driven by phylogenetically distant bacterial groups. Only Bray–Curtis dissimilarity was used in the case of metagenomic prediction. In order to account for uneven sequencing depth among samples (mean per sample coverage ± SE = 15773 ± 487, range = 1112–102922), OTU and predicted KEGG counts were converted to sample-specific proportion (as recommended by McMurdie and Holmes, 2014) prior to calculation of weighted UniFrac and Bray–Curtis dissimilarity. As this form of data normalization was not applicable for the purposes of OTU absence vs. presence analysis, we rarefied the OTU data in order to achieve the same sequence coverage per sample (i.e., corresponding to minimal sequencing depth) prior to calculation of Jaccard and unweighted UniFrac dissimilarity. Principal coordinate analysis (PCoA) and distance-based MANOVA (adonis function, vegan R package) were applied to assess whether there was any difference in FM composition between adult vs. juveniles and whether FM composition changed with juvenile age-class. We used betadisper (vegan R package) to test whether interindividual variation in FM composition differed between adults and juveniles. Those OTUs and KEGGs driving FM differences among adults vs. juveniles and among juvenile age-classes through changes in abundance were identified using DESeq2 (Love et al., 2014). As adonis, betadisper, and DESeq2 are unable to effectively account for pseudo replication due to repeat sampling of the same individual through random effects, we selected a single sample per adult and juvenile collected in 2014 at random for the purpose of these analyzes. This form of data reduction produced no hint of systematic bias or any significant decrease in statistical power; adonis and betadisper giving the same results for the complete and reduced datasets, with a high degree of correlation between the DESeq2-based log_2_ fold changes in the reduced and full datasets (Pearson’s *r* = 0.751 for OTU abundance and 0.879 for predicted KEGG abundance, *p* < 0.0001 in both cases).

We also explored co-occurrence patterns between OTUs in adults and those in individual juvenile age-classes using a recently developed version of the checkerboard score index, the nc.score (Schwager et al., 2014), on individual OTU pairs. Association between OTUs was considered significant if the corresponding *q* value (Storey and Tibshirani, 2003) was <0.05. Significant associations were used for construction of a co-occurrence network, as implemented in qgraph (Epskamp et al., 2012). Poisson generalized linear mixed effect models (GLMM; fitted using lme4), with number of significant associations per OTU as the response variable and OTU taxonomic assignation to class level as random intercept, were used to identify taxa that were more or less likely to be involved in co-occurrence or co-avoidance associations when compared to the whole FM baseline. To account for overdispersion, individual-level (i.e., OTU-specific) random effects were also included into these models. In order to meet the requirements of computational resources (24G RAM) and maintain a reasonable number of multiple tests, we filtered out those OTUs whose log_10_ scale variance for relative abundance among samples was > -8 prior to calculation (Bourgon et al., 2010).

A series of permutation-based tests were run to assess whether dissimilarity among samples corresponding to the same adult or young individual, the same nest, breeding colony or breeding season was lower than that among samples that did not match these categories. First, the average difference in ecological dissimilarity between corresponding groups (e.g., dissimilarity for the same individual vs. a different individual) was calculated and the non-parametric Cliff’s d index was used to estimate the effect size. By reshuffling dissimilarity values between sample pairs, we determined the distribution of differences expected under null hypothesis validity. The null distribution was then used for calculation of *p*-values. The subset of dissimilarities irrelevant for a given comparison and/or capable of biasing any observed difference was excluded prior to calculation. We also carefully specified blocking variables that define permutation constrains (a.k.a. strata) in order to obtain an unbiased null distribution (see details in **Data Sheet 1**). The same principle was used to test whether there was a higher similarity than expected among offspring vs. social parents.

To assess the contribution of individual OTUs to temporal stability at the community-wide level, we repeated the previous analysis for Bray–Curtis dissimilarity calculated for each of the FM OTUs. This dissimilarity metric was selected as proportion-based dissimilarities exhibit higher discriminatory power in whole-community analyses (see below). Furthermore, it is technically not possible to calculate UniFrac dissimilarity for individual OTUs. Each OTU was assumed to contribute significantly to FM stability at a *q* value threshold of ∼0.05. Following the procedures described in Bourgon et al. (2010), OTUs exhibiting low variation in abundance among samples were excluded from the dataset prior to multiple testing correction.

## Results

### General Description of Barn Swallow FM

Sequencing data comprised 7.8 million high quality paired-end reads, with reads clustered in 6222 OTUs, of which 71.4% (represented by 87.5% high quality reads) were classified to family and 45.9% (represented by 66.7% high quality reads) up to genus level. Coverage indices calculated for individual samples indicated that our data captured the vast majority of FM diversity (mean Good’s coverage ± SE = 0.994 ± 0.0003, range = 0.950–0.999).

**FIGURE 1:**
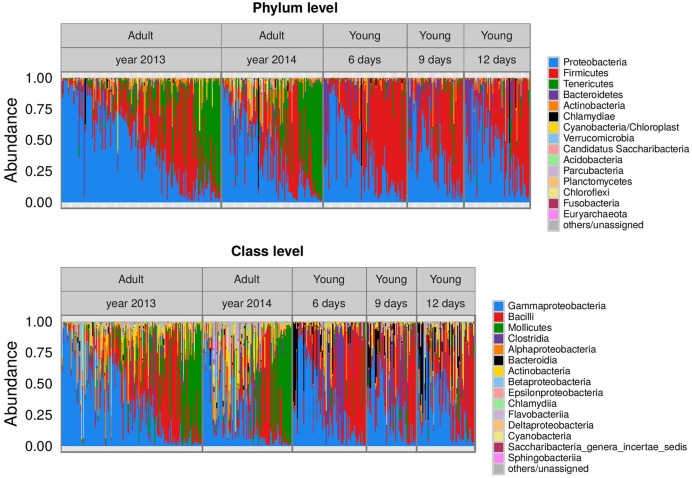
**Barplots indicating barn swallow fecal microbiota (FM) taxonomic composition.** Mean proportion of reads for the 15 dominant bacterial phyla and classes detected in juvenile and adult barn swallow samples. Samples are organized according to age category (in the case of juveniles) and year of sampling (in the case of adults). Sample order within facetes correspond to scores for the first principal coordinate analysis (PCoA) axis calculated based on weighted UniFrac.

The dataset included 36 Eubacterial phyla and one Archaeal phylum (Euryarchaeota). Barn swallow FM was dominated by Proteobacteria (mean ± SE of reads = 39.6 ± 1.4% reads, range = 45.5–98%), Firmicutes (34.7 ± 1.4%, range = 13.2–99.1%), Tenericutes (12.7 ± 1.1%, range = 0–96.0%), Bacteroidetes (6.3 ± 0.7%, range = 0–92.2%), and Actinobacteria (4.4 ± 0.4%, range = 0–48.7%), with other phyla present at much lower frequencies (at average <1% reads). At lower taxonomic levels, Proteobacteria were predominantly represented by unclassified Enterobacteriaceae (10.7% of all high quality reads), *Serratia* (5.1%), *Pantoea* (3.3%), *Aeromonas* (2.4%), *Pseudomonas* (1.1%), and *Rickettsia* (1.4%). The most abundant Firmicutes genera were *Enterococc*us (7.7% of all high quality reads), *Catellicoccus* (*5.8%), Lactobacillus* (4.3%), and *Lactococcus* (1.3%*). Dysgonomonas* (4.2% of reads) was the dominant genus from the phylum Bacteroidetes, while the phylum Tenericutes was predominantly represented by *Mycoplasma* (9.6% of reads) and *Ureaplasma* (1.6%). The plot for taxonomic assignment of FM indicated pronounced variation at the inter-individual level (**Figure 1**) as well as consistent differences among adult and juveniles (detailed in the next section). A more comprehensive description of FM taxonomic content is provided in **Supplementary Table S2**.

### FM Changes during Ontogeny

**FIGURE 2:**
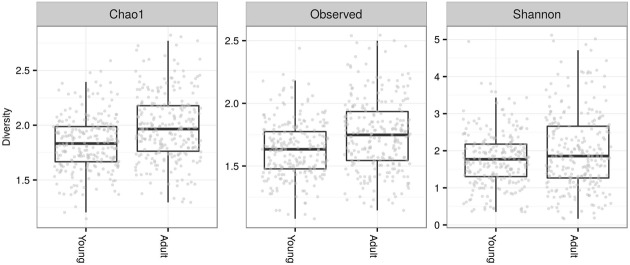
**Alpha diversity of FM in juveniles vs. adults.** Number of observed operational taxonomic units (OTUs) and Chao1 estimates were log_10_ scaled, whereas Shannon diversities were not transformed.

Fecal microbiota alpha diversity in terms of observed OTUs was nearly two-times higher in adults (mean ± SE of observed OTUs prior to rarefaction; adults = 222 ± 11.4, young = 140 ± 5.4). The contrast in alpha diversity between adults and young was significant for all alpha diversity indices calculated following rarefaction-based normalization of the OTU table (LME: ΔDF = 1, χ^2^ = 21.832, *p* < 0.0001 for number of observed OTU; ΔDF = 1, χ^2^ = 29.85, *p* < 0.0001 for Chao1 estimate; ΔDF = 1, χ^2^ = 6.944, *p* = 0.008 for Shannon diversity; **Figure 2**).

**FIGURE 3:**
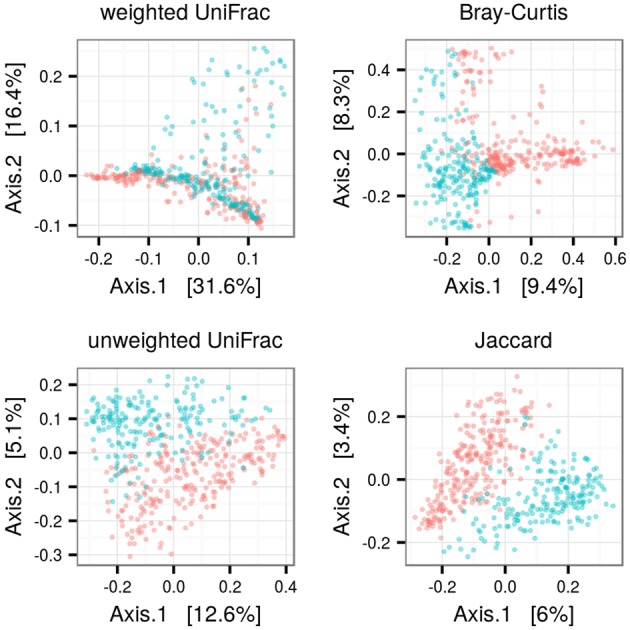
**Difference in FM composition among adult and juvenile barn swallows.** Principle coordinate analysis for ecological dissimilarity among FM samples of juvenile (blue) and adult (red) barn swallows, with proportion of FM variation associated with individual axes indicated.

PCoA and adonis revealed consistent differences in FM composition among adults vs. young (**Figure 3**). Betadisper revealed lower inter-individual FM variation in juveniles for all dissimilarity index types. This difference was more pronounced in OTU presence vs. absence based methods (Jaccard and unweighted UniFrac; **Table 1**) than those based on relative abundance (**Table 1**). PCoA for predicted metagenomes indicated only slight differentiation among adults and juveniles (**Data Sheet 1**), though the differentiation was significant in adonis (**Table 1**). As in the OTU-based analyses, inter-individual variation in predicted metagenomes was higher in adults (**Table 1**).

**Table 1:** Divergence between adult vs. juvenile barn swallow fecal microbiota (FM).

		Adonis						Betadisper			
			
Input data	Effect	Df	SS	MSS	*F*	*R*^2^	*p*	SS	MSS	*F*	*p*
OTUs: weighted UniFrac	Adults vs. young	1	0.331	0.331	11.603	0.067	0.001	0.012	0.012	5.204	0.024
	Residuals	161	4.596	0.029		0.933		0.359	0.002		
OTUs: unweighted UniFrac	Adults vs. young	1	2.575	2.575	11.253	0.065	0.001	0.147	0.147	29.346	<0.001
	Residuals	161	36.835	0.229		0.935		0.804	0.005		
OTUs: Bray–Curtis	Adults vs. young	1	4.015	4.015	10.250	0.060	0.001	0.030	0.030	7.346	0.007
	Residuals	161	63.065	0.392		0.940		0.654	0.004		
OTUs: Jaccard	Adults vs. young	1	3.142	3.142	8.527	0.050	0.001	0.058	0.058	33.110	<0.001
	Residuals	161	59.331	0.369		0.950		0.282	0.002		
KEGGs: Bray–Curtis	Adults vs. young	1	0.070	0.070	5.949	0.036	0.003	0.015	0.015	6.166	0.014
	Residuals	161	1.902	0.012		0.964		0.401	0.002		


*ANOVA tables for adonis and betadisper models testing differences in FM composition and inter-individual variation among adults and juveniles. Models were fitted for four ecological distance types (weighted and unweighted UniFrac, Bray–Curtis and Jaccard) for the OTU dataset. Shown are treatment and residual degrees of freedom (Df), sum of squares (SS), mean sum of squares (MSS), *F*-statistic values (*F*), probability values (*p*) and proportion of explained variance (*R*^*2*^).*

**FIGURE 4:**
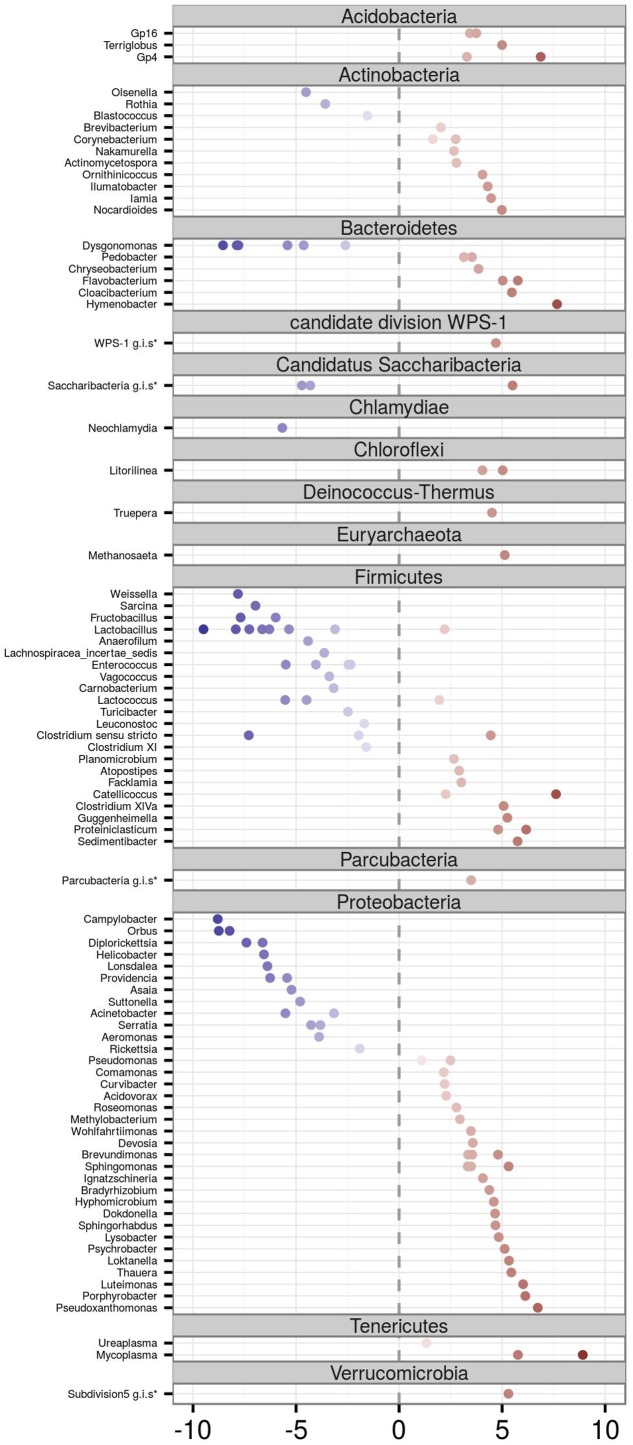
**Differential abundance analysis for FM OTUs of adult vs. juvenile barn swallows.** Only those OTUs whose abundance varied significantly among adults and juveniles according to DESeq2 analyses are shown. Position on the horizontal axis corresponds to its log_2_ fold change. Negative (blue) and positive values (red) indicate a higher OTU abundance in juveniles and adults, respectively. Genus identity is determined by labels on the left side of the plot. OTUs are sorted according to their Phylum assignment. (^∗^g.i.s. = *genera incertae sedis*).

DESeq2 analysis identified 213 OTUs (represented by 69.5% high quality reads) in which abundance varied between adults vs. juveniles, with 81 OTUs overrepresented in juveniles and 132 in adults (**Figure 4**). OTUs corresponding to the phyla Acidobacteria (genera *Gp16*, *Gp4*, and *Terriglobus*), Tenericutes (genera *Mycoplasma* and *Ureaplasma*), Verrucomicrobia, Parcubacteria, Deinococcus-Thermus (genus *Truepera*), Chloroflexi (genus *Litorilinea*), and Euryarchaeota (genus *Methanosaeta*) were more abundant in adults. The same was true for most Bacteroidetes OTUs (genera *Hymenobacter*, *Cloaci-bacterium*, *Flavobacterium*, *Chryseobacterium*, and *Pedobacter*) as well as most Actinobacteria OTUs (corresponding to the genera *Nocardioides*, *Iamia*, *Ilumatobacter*, *Ornithinicoccus*, *Actinomycetospora*, *Nakamurella*, and *Corynebacterium*). Juvenile FM was characterized by an increase in the abundance of Chlamydiae (genus *Neochlamydia*) and several OTUs corresponding to genus *Dysgonomonas* (phylum Bacteroidetes) and *Olsenella*, *Rothia*, and *Blastococcus* (all belonging to the phylum Actinobacteria). The two dominant barn swallow FM phyla, Proteobacteria and Firmicutes, harbored a mix of OTUs that were overrepresented in either adults or juveniles. In the case of Proteobacteria, *Pseudoxanthomonas*, *Porphyrobacter*, *Luteimonas*, or *Thauera* exhibited a considerable increase in abundance in adults (log_2_ fold change > 5), while *Campylobacter*, *Orbus*, *Helicobacter*, *Lonsdalea*, or *Providencia* were more abundant in young. In the case of Firmicutes, *Sedimentibacter*, *Proteiniclasticum, Guggenheimella*, or *Catellicoccus* were overrepresented in adults, while abundance of *Weissella*, *Sarcina*, *Fructobacillus*, and most *Lactobacillus* OTUs increased in young.

Consistent with the pronounced differences observed at the OTU level, the abundance of 150 KEGG categories (DESeq2) also varied significantly between adults and juveniles, though the effect-size of these changes was low in most cases. For a summary of 34 KEGGs for which abundance varied considerably between adults vs. juveniles (absolute value of log_2_ fold change > 1), see **Data Sheet 1**. FM diversity tended to increase with increasing juvenile age [LME; observed OTUs (log10 transformed): slope = 1.140e-02 ± 6.629e-03, ΔDF = 1, χ^2^ = 3.877, *p* < 0.05; Chao1 (log10 transformed): slope = 0.057 ± 0.0188, ΔDF = 1, χ^2^ = 20.492, *p* = 0.0876; Shannon diversity: slope = 1.295 ± 0.05692, ΔDF = 1, χ^2^ = 8.946, *p* = 0.0028]. On the other hand, age-dependent changes in juvenile FM composition were not significant for weighted and unweighted UniFrac according to adonis (*p* > 0.1, *R*^2^ < 0.02), while only slight age-dependent differences were observed if using Jaccard and Bray–Curtis dissimilarity (*p* < 0.05, *R*^2^ = 0.014 and *p* < 0.01, *R*^2^ = 0.013, respectively). Inter-individual variation in FM did not change with juvenile age (betadisper: *p* > 0.7 in all cases) and no change in OTU abundance with juvenile age was detected using DESeq2 analysis. Similarly, no significant differences were observed among predicted metagenomes in juvenile age-classes (*p* > 0.2 for both adonis and betadisper).

**FIGURE 5:**
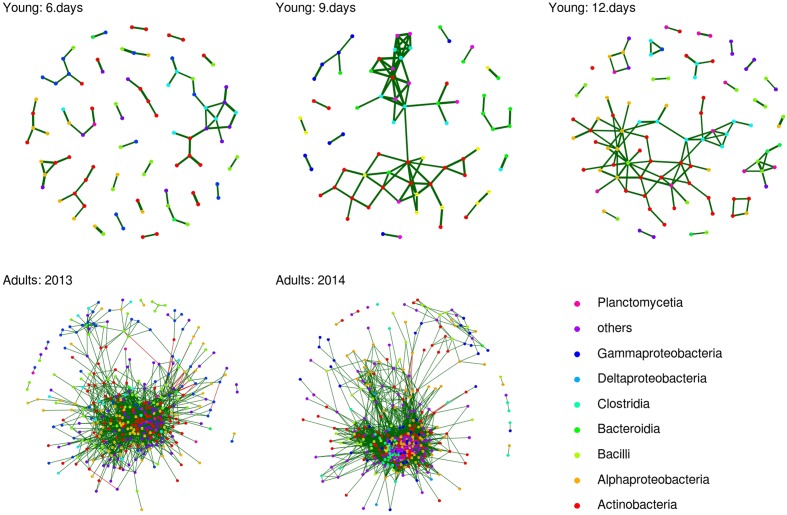
**Operational taxonomic unit co-occurrence network for adult and juvenile barn swallow FM.** OTU co-occurrence network based on nc.scores for individual age-classes and for adults. Only those nc.scores passing multiple testing corrections (*q* < 0.05) are shown. Green and red lines indicate positive and negative co-associations, respectively. Class-level taxonomic assignations of individual OTUs included in the analysis are highlighted in different colors.

As there was significant excess of positive nc.scores for adults and all juvenile age-classes (Wilcoxon one sample test: *p* < 0.001 in all cases), FM structure appeared to be driven predominantly by co-occurrence rather than co-avoidance interactions. Furthermore, nc.score values exhibited highly significant correlations across all juvenile age-classes and adults (Mantel’s test: correlation coefficient range = 0.338–0.391, *p* < 0.0001 in all cases) and in adults sampled in 2013 vs. 2014 (Mantel’s *r* = 0.481, *p* < 0.0001), suggesting that among-OTU co-occurrence/co-avoidance interactions varied little over time and were independent of host age. After filtering out non-significant nc.scores, average number of co-occurrence or co-avoidance links per OTU was 0.137, 0.207, and 0.237 for six day-, nine day- and 12 day-old juveniles, respectively, and 3.437 and 5.242 for adults sampled in 2013 and 2014, respectively (**Figure 5**). The number of interactions per OTU was positively correlated with relative log-transformed OTU abundance (*p* > 0.0001 in all cases). After statistical control for this confounding effect, GLMM-based random effect estimates indicated that the number of significant per-OTU interactions was increased in Actinobacteria and Alphaproteobacteria bacteria consistently across all juvenile age-classes. The same also held true for Clostridia, with the exception of co-occurrence analysis on adults sampled in 2013 (see **Data Sheet 1**).

### Temporal Invariance of FM and Divergence among Nests and Breeding Colonies

**FIGURE 6:**
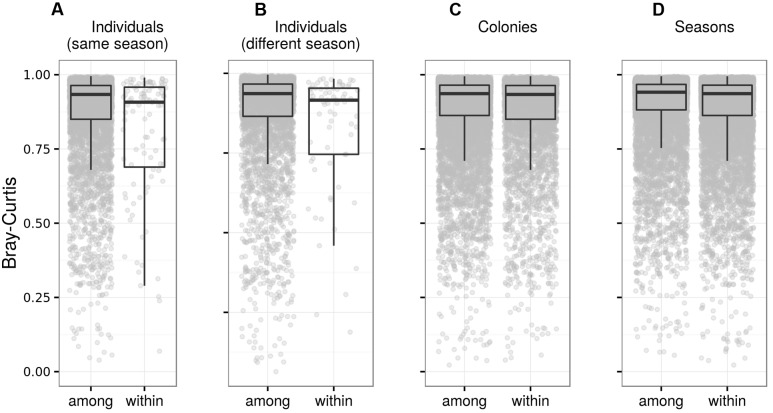
**Fecal microbiota differentiation in adult barn swallows.** Box plots for Bray–Curtis dissimilarity among FM samples from the same vs. different individuals sampled during **(A)** the same breeding season and **(B)** different breeding seasons, **(C)** dissimilarities among FM samples from different individuals sampled during the same breeding season in the same vs. different colony, and **(D)** dissimilarities among FM samples from non-identical individuals sampled in the same breeding colony during the same vs. different breeding season. Comparisons based on other types of ecological dissimilarity are available in the **Data Sheet 1**.

Bray–Curtis and weighted UniFrac similarity among samples from the same adults collected within individual breeding seasons was higher than that among samples from different adults (**Table 2**; **Figure 6**; **Data Sheet 1**), providing evidence for temporal stability in adult FM composition. When using unweighted UniFrac or Jaccard dissimilarity for the same test, however, we found no support for within-individual FM composition stability (**Table 2**; **Data Sheet 1**). Dissimilarity at within-individual and within-season time-scales tended to increase with increasing time-lag between collection of the corresponding samples (average time-lag ± SE = 34.6 ± 1.7 days) in the case of unweighted UniFrac (LME: ΔDF = 1, χ^2^ = 4.526, *p* = 0.0334), Jaccard (LME: ΔDF = 1, χ^2^ = 7.731, *p* = 0.0054) and Bray–Curtis dissimilarity (LME: ΔDF = 1, χ^2^ = 4.560, *p* = 0.0327), after box–cox transformation of the response variable. This relationship was not significant for weighted UniFrac (LME: ΔDF = 1, χ^2^ = 0.404, *p* = 0.5253). At the between-season level, FM profiles corresponding to the same adult only exhibited higher similarity than expected by chance based on Bray–Curtis dissimilarity (**Table 2**). Temporal stability of predicted adult metagenome content was significant at the within-season time-scale, but not at the between-season time-scale (**Table 2**). Higher similarity between adults within breeding colonies than that between breeding colonies was associated with a very low effect size, despite being significant for all community distances types. Similarly, between-year variation in FM was associated with a considerable effect size in the case of Jaccard dissimilarity only (**Table 2**).

**Table 2:** Effect of individual identity, breeding colony, and year on the divergence of adult barn swallow FM.

	Within vs. among individuals (within years)				Within vs. among individuals (among years)			
		
	Observed diff.	95% CI range	*p*	Cliff’s *d*	Observed diff.	95% CI range	*p*	Cliff’s *d*
OTUs: weighted UniFrac	**0.024**	-**0.015~0.013**	**0.001**	**0.138**	0.011	-0.016~0.016	0.118	0.046
OTUs: unweighted UniFrac	-0.004	-0.015~0.011	0.591	-0.018	0.003	-0.014~0.015	0.42	-0.001
OTUs: Bray–Curtis	**0.079**	-**0.022~0.027**	** <0.001**	**0.194**	**0.056**	-**0.028~0.028**	**0.001**	**0.195**
OTUs: Jaccard	-0.002	-0.007~0.008	0.692	-0.011	0.007	-0.009~0.009	0.077	0.061
KEGGs: Bray–Curtis	**0.015**	-**0.014~0.011**	**0.015**	**0.101**	**0.018**	-**0.014~0.015**	**0.026**	**0.157**

	**Within vs. among colonies**				**Within vs. among years**			
		
	**Observed diff.**	**96% CI range**	***p***	**Cliff’s d**	**Observed diff.**	**96% CI range**	***p***	**Cliff’s d**

OTUs: weighted UniFrac	**0.008**	-**0.002~0.002**	**<0.001**	**0.013**	**0.008**	-**0.002~0.002**	**<0.001**	**0.061**
OTUs: unweighted UniFrac	**0.012**	-**0.002~0.002**	**<0.001**	**0.049**	**0.012**	-**0.002~0.002**	**<0.001**	**0.093**
OTUs: Bray–Curtis	**0.008**	-**0.004~0.004**	**<0.001**	**0.029**	**0.008**	-**0.003~0.003**	**<0.001**	**0.051**
OTUs: Jaccard	**0.01**	-**0.001~0.001**	**<0.001**	**0.072**	**0.01**	-**0.001~0.001**	**<0.001**	**0.127**
KEGGs: Bray–Curtis	**0.003**	-**0.002~0.002**	**<0.001**	**0.009**	**0.003**	-**0.002~0.002**	**<0.001**	**0.028**


*Results of permutation-based tests on adult FM samples comparing dissimilarity among samples from (A) the same vs. different individuals sampled during the same breeding season and the same colony, (B) the same vs. different individuals sampled during different breeding seasons and in the same breeding colony, (C) different individuals sampled in the same vs. different breeding colony during the same breeding season, and (D) different individuals sampled in the same breeding colony during the same vs. different breeding season. For OTU data, analyses were run on four dissimilarity index types (weighted and unweighted UniFrac, Bray–Curtis and Jaccard). Shown are observed dissimilarity difference values, 95% confidence intervals of the corresponding permutation-based null distribution, permutation-based probability values and effect size estimates (Cliff’s *d*). Significant results are in boldface.*

We identified 63 OTUs in the adult FM for within-season analysis and 118 OTUs for between-season analysis (represented by 30 and 20% of high-quality reads, respectively) where abundance exhibited significantly lower variation within individuals than between individuals. At the FM community level, signatures of temporal stability increased after exclusion of all but these OTUs from the dataset (mean difference between within- vs. between-individual dissimilarities = 0.125 and 0.010, Cliff’s *d* = 0.282 for within-season and 0.275 for between-season time-scales, *p* < 0.001 in both cases; **Data Sheet 1**). Seventeen OTUs, assigned to Enterobacteriaceae, *Acinetobacter*, *Corynebacterium*, *Dysgonomonas*, *Tsukamurella*, *Dietzia*, *Mycoplasma*, *Streptococcus*, *Catellicoccus*, and *Lactobacillus*, exhibited significant temporal consistency both within and between seasons.

**FIGURE 7:**
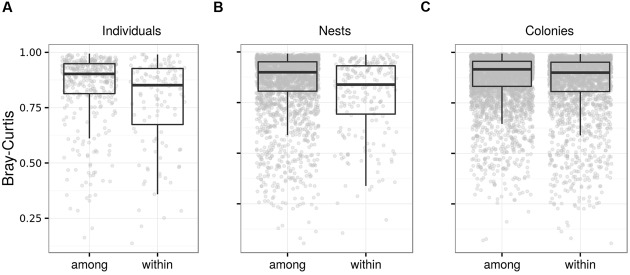
**Fecal microbiota differentiation in juvenile barn swallows.** Box plots for Bray–Curtis dissimilarity among FM samples from **(A)** identical vs. different individuals from the same nest corresponding to different age-classes, **(B)** different individuals from identical vs. different nests placed in the same breeding colony and corresponding to the same age-classes, and **(C)** individuals from different nests that were placed in the same vs. different breeding colony. Comparisons based on other types of ecological dissimilarity are available in the **Data Sheet 1**.

Community-wide juvenile FM temporal consistency was significant based on weighted UniFrac and Bray–Curtis dissimilarity calculated for OTU; however, there was no hint of temporal consistency based on absence vs. presence dissimilarities and KEGG predictions (**Table 3**; **Figure 7**). In addition, OTU-centered analysis identified only 16 low-abundance OTUs (represented by 1.4% high quality reads) that contributed to FM stability in juveniles. Differences in Bray–Curtis dissimilarity for sample pairs corresponding to the same vs. different juvenile were comparable when calculated for either the whole FM (**Table 3**) or for the FM subset comprising the 16 OTUs exhibiting signs of temporal stability (mean difference between within- vs. between-individual dissimilarity = 0.0765, Cliff’s *d* = 0.125, *p* < 0.01; **Data Sheet 1**). FM similarity for juveniles of the same age at the within-clutch level was higher than that of juveniles raised in different clutches, both for OTU and predicted KEGG data and irrespective of dissimilarity index used (**Table 3**; **Figure 7**). The within-clutch similarity effect-size decreased considerably when running this analysis on FM samples from individuals of different age (Cliff’s *d* < 0.05 in all cases). We also observed higher FM similarity within breeding colonies than between breeding colonies (**Table 3**; **Figure 7**), though the effect-size was low in all cases (Cliff’s *d* < 0.1).

**Table 3:** Effect of individual identity, clutch identity, and breeding colony on FM divergence in juvenile barn swallows.

	Within vs. among individuals				Within vs. among nests			
		
	Observed diff.	95% CI range	*p*	Cliff’s *d*	Observed diff.	95% CI range	*p*	Cliff’s *d*
OTUs: weighted UniFrac	0.027	-0.002~0.025	0.023	0.159	**0.022**	-**0.007~0.012**	**0.001**	**0.142**
OTUs: unweighted UniFrac	**0.005**	-**0.013~0.015**	**0.323**	**0.011**	**0.022**	-**0.015~0.007**	**<0.001**	**0.144**
OTUs: Bray–Curtis	0.07	0.004~0.057	0.006	0.236	**0.064**	-**0.015~0.02**	**<0.001**	**0.22**
OTUs: Jaccard	**0.003**	-**0.008~0.011**	**0.37**	**0.018**	**0.03**	-**0.008~0.006**	**<0.001**	**0.237**
KEGGs: Bray–Curtis	0.004	-0.002~0.014	0.684	0.042	**0.016**	-**0.003~0.009**	**0.001**	**0.184**

	**Within vs. among localities**							
		
	**Observed diff.**	**96% CI range**	***p***	**Cliff’s *d***				

OTUs: weighted UniFrac	0.002	-0.004~0.003	0.101	0.017				
OTUs: unweighted UniFrac	**0.013**	-**0.004~0.004**	**<0.001**	**0.083**				
OTUs: Bray–Curtis	**0.026**	-**0.006~0.006**	**<0.001**	**0.084**				
OTUs: Jaccard	**0.009**	-**0.002~0.003**	**<0.001**	**0.094**				
KEGGs: Bray–Curtis	-0.003	-0.002~0.002	0.977	-0.028				


*Results of permutation-based tests comparing dissimilarity among juvenile FM samples collected from (A) the same vs. different individuals in the same nest, (B) different individuals from the same vs. different nests located in the same breeding colony, and (C) individuals from different nests in the same vs. different breeding colony. For OTU data, analyses were run on four dissimilarity index types (weighted and unweighted UniFrac, Bray–Curtis and Jaccard). Only Bray–Curtis dissimilarity was used for predicted KEGG categories. Shown are observed dissimilarity difference values, 95% confidence intervals of permutation-based null distribution, permutation-based probability values and effect size estimates (Cliff’s *d*). Significant results are in boldface.*

### FM Similarity between Offspring and Their Parents

**FIGURE 8:**
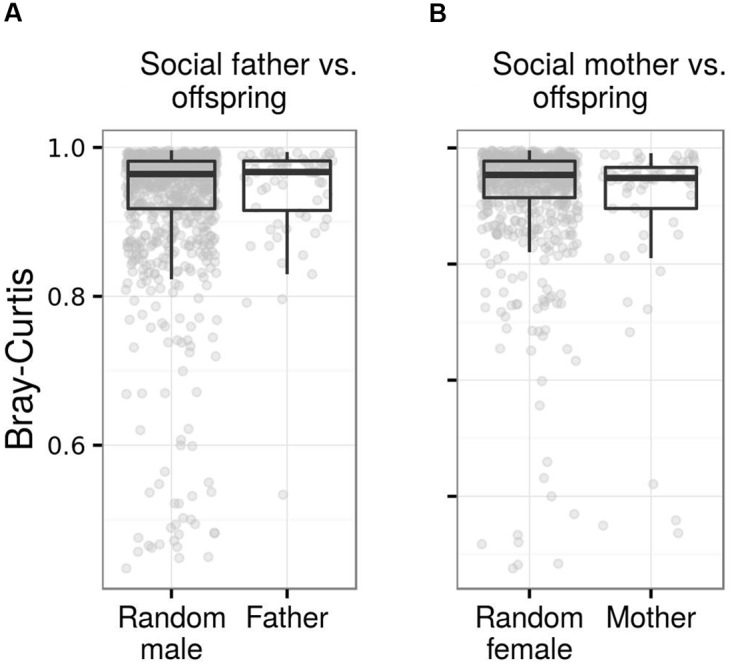
**Fecal microbiota similarity between young barn swallows and their social parents.** Box plots for Bray–Curtis dissimilarity among juveniles and **(A)** their social fathers vs. non-social adult males and **(B)** social mothers vs. non-social adult females. Comparisons based on other types of ecological dissimilarity are available in the **Data Sheet 1**.

While juvenile FM showed greater similarity to social mothers than random adult females when using weighted UniFrac or Bray–Curtis as a measure of FM divergence, we observed no such effect when using absence vs. presence dissimilarities. We also observed no effect of social father on the FM composition of its offspring (**Table 4**; **Figure 8**; **Data Sheet 1**). These results remained unchanged after exclusion of extra-pair (*n* = 9) young from social father vs. offspring and parasitic young (*n* = 3) from social mother vs. offspring comparisons. Offspring vs. mother or father dissimilarity did not vary with juvenile age (LME: *p* > 0.2 in all cases). There was also no association between time lag in social mother vs. offspring FM sampling and FM similarity between mother and offspring (LME, *p* > 0.1 in all cases). On the other hand, social father vs. offspring Bray–Curtis and weighted UniFrac dissimilarity tended to increase with increasing time-lag between the two samples (LME, Bray–Curtis: ΔDF = 1, χ^2^ = 6.136, *p* = 0.0133; weighted UniFrac: ΔDF = 1, χ^2^ = 3.5994, *p* = 0.0570), though this effect was non-significant when using unweighted UniFrac and Jaccard dissimilarity (*p* < 0.1 in both cases). Similarity in the composition of predicted metagenomes among juveniles and social mothers or fathers was not higher than expected by chance (**Table 4**).

**Table 4:** Barn swallow parent vs. offspring similarity.

	Father vs. offspring similarity				Mother vs. offspring similarity			
		
	Observed diff.	95% CI range	*p*	Cliff’s *d*	Observed diff.	95% CI range	*p*	Cliff’s *d*
OTUs: weighted UniFrac	-0.004	-0.017~0.017	0.631	-0.016	**0.026**	-**0.022~0.022**	**0.031**	**0.148**
OTUs: unweighted UniFrac	0.01	-0.016~0.016	0.188	0.074	-0.005	-0.024~0.024	0.616	-0.038
OTUs: Bray–Curtis	-0.005	-0.016~0.018	0.639	-0.022	**0.052**	-**0.028~0.034**	**0.013**	**0.171**
OTUs: Jaccard	0.006	-0.01~0.01	0.194	0.079	0.002	-0.013~0.013	0.4	0.035
KEGGs: Bray–Curtis	0.005	-0.019~0.018	0.328	0.003	-0.007	-0.017~0.017	0.724	-0.104


*Results of permutation-based tests comparing dissimilarity among parental vs. offspring FM composition. Analyses were run separately for offspring vs. mother and offspring vs. father pairs. For OTU data, analyses were run on four dissimilarity index types (weighted and unweighted UniFrac, Bray–Curtis and Jaccard). Only Bray–Curtis dissimilarity was used for predicted KEGG categories. Shown are observed value dissimilarity differences, 95% confidence intervals of permutation-based null distribution, permutation-based probability values and estimates of effect size (Cliff’s *d*). Significant results are in boldface.*

## Discussion

### Age-Dependent Variation in FM Structure

Our data revealed pronounced differences in FM structure between adult and juvenile barn swallows. Adult FM alpha diversity was nearly two-times higher than that of juveniles, which, together with a slight increase in alpha diversity with juvenile age, implies that FM is gradually colonized by bacteria from external sources during the nestling period. On the other hand, six day-old juveniles had already been colonized by a rich FM consortia and the FM taxonomic composition in juveniles did not show any great variation with age. Hence, initial establishment of juvenile-specific FM appears to take place very soon after hatching and there is no evidence for a gradual succession toward adult-like microbiota due to newly invading FM species over the nestling period. In humans and other mammalian taxa, dramatic shifts in FM composition coincide with the transition between the pre- and post-weaning period due to associated nutritional changes (Schloss et al., 2012; Bäckhed et al., 2015; Mach et al., 2015). The effect of diet on mammalian host FM has also been demonstrated in numerous experimental and comparative studies (Ley et al., 2008; David et al., 2014). We propose, however, that diet only has a limited effect on the differences in FM between adult and juvenile in barn swallow. While slight differences in adult vs. juvenile barn swallow diet have previously been reported, both these cohorts forage exclusively on taxonomically similar groups of flying insects (Turner, 1980). As a result, the potential effect of diet composition on FM is much more restricted in our study compared to research focused on omnivorous hosts. Moreover, our data on more than 50 passerine species with contrasting foraging specializations indicates that diet only has a negligible effect on interspecific variation in FM composition in this group (Kropáčková et al., unpublished data). Age-specific differences in gut physiology, morphology and diet digestion could also affect FM variation between adults vs. juveniles, especially as these undergo dramatic development after hatching in passerines and other bird taxa. Such changes include a gradual increase in relative gut mass and surface area, proliferation of enterocytes and mucous-secreting goblet cells (reviewed in Perry, 2006) and an increase in production of digestive enzymes (Caviedes-Vidal and Karasov, 2001), all of which increase digestion efficiency with host age (Karasov, 1990). Similarly, adaptive immunity and some components of innate immunity, are not fully developed in juveniles (Killpack et al., 2013), implying a reduced capability for management of associated microbiota in juveniles. Alternatively, FM variation between adults vs. juveniles could also be partly affected by age-dependent differences in the sources of bacteria invading the host’s body. While the most likely source for juvenile FM are bacteria in the nest material or those acquired via parental transfer (González-Braojos et al., 2012a), adults are exposed to a much more diverse pool of environmental bacteria, both at the breeding ground and during the migration.

Taxonomic differences between adult and juvenile FM may provide a more mechanistic insight into those processes shaping FM differences between the two cohorts. Juvenile FM, for example, is characterized by an increase in the abundance of OTUs corresponding to lactic acid bacteria (LAB; genera *Lactobacillus*, *Leuconostoc*, *Lactococcus*, *Carnobacterium*, *Enterococcus*, *Vagococcus*, *Weissella*, and *Olsenella*) and family Enterobacteriaceae (genera *Serratia*, *Lonsdalea*, and *Providencia*). LAB, and many Enterobacteriaceae, prefer energy-rich substrates and are capable of fermenting carbohydrates under anoxic conditions (Kandler, 1983). Their presence in juveniles, therefore, may reflect reduced production of digestive enzymes specific for this substrate (Caviedes-Vidal and Karasov, 2001). Presence of some LAB is believed to be generally beneficial as they stimulate the host’s immune system and produce metabolites involved in the maintenance of gut microbiota homeostasis (Ljungh and Wadström, 2006). Further, some LAB species have been reported as contributing to the host’s energy balance via improved feed conversion (Abe et al., 1995). On the other hand, while the specific effects of LAB and Enterobacteriaceae on passerine hosts are still poorly understood, it is thought that some may trigger negative consequences, including reduced growth rates and competition with the host for energy resources (González-Braojos et al., 2012b). Unlike juvenile FM, adult FM was enriched with bacteria utilizing relatively complex substrates, e.g., *Sedimentibacter*, *Clostridium* cluster XIVa, and *Proteiniclasticum* (family Clostridiaceae) or Flavobacterium and Chryseobacterium (family Flavobacteriaceae). In juveniles, we also observed an increase in the abundance of OTUs corresponding to taxa that may be associated with pathogenic or other detrimental effects on avian hosts. These include genera *Helicobacter* (Harbour and Sutton, 2008), *Campylobacter* (Benskin et al., 2015), *Rickettsia* and *Diplorickettsia* (Ritchie et al., 1994), or *Suttonella* (Kirkwood et al., 2006), which may be associated with lowered capacity of the juvenile’s immune system to cope with detrimental bacterial invaders (Killpack et al., 2013). Potential pathogens that were more abundant in adult FM included genera *Mycoplasma* and *Ureaplasma* (Sumithra et al., 2013). These bacteria often invade the urogenital tract of birds; hence, we speculate that their presence is associated with changes occurring in the urogenital tract during the breeding season or with sexual contact between colony members (Kreisinger et al., 2015b). We also detected an increase in OTUs of the family Xanthomonadaceae (genera *Wohlfahrtiimonas*, *Lysobacter*, *Luteimonas*, *Pseudoxanthomonas*, *Dokdonella*, and *Ignatzschineria*) in adult FM, as well as several Alphaproteobacterial OTUs (for example genera *Devosia*, *Hyphomicrobium*, *Porphyrobacter*, *Loktanella*) that are likely to be of environmental origin, suggesting a larger effect on adult FM of bacteria from environmental pools. However, as these taxa include both opportunistic pathogens and species harboring potentially important functions for vertebrate hosts, including defense against pathogens (Boutin et al., 2012), we cannot exclude their functional significance in barn swallow microbiota.

According to PICRUSt, the adult FM was enriched with a number of KEGGs that may be involved in interactions with the host’s immunity system or with other FM members. Bacterial production of melanin, for example, can be associated with scavenging of superoxide radicals, which probably makes bacteria more resistant to the oxygen burst induced by the host’s immune system (Plonka and Grabacka, 2006). Similarly, KEGG categories involved in the synthesis of metabolites that have putatively antibacterial and antifungal properties, such as sesquiterpenoid and macrolides, may promote competitive interactions with other FM members, thereby contributing to increased adult FM complexity, as discussed below. PICRUSt analysis also indicated that the adult FM may have a larger effect on host energy balance than that of juveniles as the abundance of those KEGGs associated with protein and carbohydrate digestion and absorption is increased. Bacterial fermentation of these substrates produces short-chain fatty acids that can be utilized as an energy source by the vertebrate host. Moreover, short-chain fatty acids are directly involved in the regulation of gut physiology and attenuate inflammatory responses (den Besten et al., 2013). The adult FM was also enriched with several KEGGs associated with ion balance (particularly: “Calcium signaling pathways” and “Endocrine and other factor-regulated calcium reabsorption”) and pathways regulated by ion concentration (e.g., “Vasopressin-regulated water reabsorption” or “Gastric acid secretion”). Overall, FM predictions indicate higher interaction complexity between the host and gut microbiota and between individual members of the gut microbiota community in adults. On the other hand, several KEGGs found at higher levels in the adult FM are not directly associated with animal hosts (e.g., Isoflavim or Betalain biosynthesis), suggesting a greater importance of environmental bacteria in the adult FM.

Interestingly, differences in FM structure between adults and juveniles were observed not only in terms of richness and composition but also at the level of OTU co-occurrence pattern, with co-occurrence networks being more complex in adults. There was a significant correlation between co-occurrence coefficients calculated for adults and for different juvenile age-classes, with Alphaproteobacteria, Actinobacteria, and Clostridia being the main drivers of co-occurrence interaction, irrespective of host age. While this suggests that the overall shape of co-occurrence interactions remains invariant in relation to host age, co-occurrence strength increased with the end of the nestling period. Significant links in co-concurrence networks are often interpreted as direct interactive associations between two bacterial taxa (Faust et al., 2012). The abundance of two OTUs may be positively correlated, for example, if one utilizes the product of the other as a metabolic substrate (reviewed in Morris et al., 2013). On the other hand, secondary metabolites in certain species may suppress proliferation of other FM members, resulting in negatively correlated abundances (Ruiz et al., 2009). Alternatively, heterogeneity in environmental factors modulating FM composition may affect inter-individual variation in the FM community. Consequently, abundance correlations among OTUs may be driven by these extrinsic sources of FM variation rather than by direct mutual OTU interaction. In line with this explanation, inter-individual variation in adult FM was higher, implying an increased potential for detection of apparent interactive links between OTUs. At the same time, Alphaproteobacteria harboring OTUs of putatively environmental origin, were identified as one of the main drivers of co-occurrence interactions. On the other hand, Actinobacterial OTUs, which commonly produce secondary metabolites involved in interactions with other gut bacteria (Riley and Wertz, 2002), and Clostridia, which have been identified as important co-occurrence drivers in human gut microbiota (Faust et al., 2012), were both involved in co-occurrence interactions in barn swallow FM more than expected by chance. This suggests that the patterns observed were at least partly driven by direct OTU vs. OTU interactions.

### Temporal Stability of FM

Fecal microbiota is shaped by a wide variety of factors, some of which presumably have a stabilizing effect on the temporal consistency of its composition (Benson et al., 2010; McKnite et al., 2012) while others contribute to rapid turnover of FM members, introducing instability into the FM community over time (Amato et al., 2013; Wang et al., 2014). Our data on a free-living passerine bird population has extended current understanding of FM stability over time, with only one other study addressing this topic in this vertebrate group, however, using a captive population (Benskin et al., 2010). We show that, for both juveniles and adults, temporal consistency in taxonomic and functional profiles at the whole FM community level exhibited a rather low effect-size, despite its statistical significance. The most striking evidence for FM temporal stability has been provided for human populations, where individual-specific FM signatures persist for several years (Faith et al., 2013; Lim et al., 2014; Salonen et al., 2014, but see David et al., 2014). A high degree of temporal invariance in human FM may be maintained partly through long-term consistency in the social micro-culture environment and associated temporal stability of biotic and abiotic factors shaping FM (Mulder et al., 1998; Edstrom and Devine, 2001; Borland et al., 2007). In comparison, the few studies focused on FM temporal stability in other mammalian species typically show rapid within-individual FM composition fluctuations (Schloss et al., 2012; Becker et al., 2015; Hoy et al., 2015; but see Stevenson et al., 2014). Current knowledge on individual FM composition over time in wild populations is very limited (Waite et al., 2014; Baxter et al., 2015; Sun et al., 2016). Nevertheless, previous studies on free-living vertebrates have revealed pronounced population-wide changes in FM due to switches in diet composition, physiological state or health and infection status (Amato et al., 2013; Kreisinger et al., 2015a; Maurice et al., 2015; Sommer et al., 2016). Hence, the relatively low temporal stability in barn swallow FM detected in our study is in general agreement with the vast majority of current studies on vertebrates. As we controlled our analysis for systematic FM variation among breeding colonies, we can assume that the slight yet significant FM consistency observed was not caused by environmental heterogeneity within the populations sampled. In addition, given the small area of the breeding colonies (several hundreds of square meters), the high environmental homogeneity within colonies and low FM differentiation between colonies, it is unlikely that any environmental variation operating at the within-colony level could contribute to temporally consistent differences in FM between individuals.

It has recently been proposed that the temporal stability in microbial communities is maintained by a subset of “conditionally rare taxa” (Shade and Gilbert, 2015) that exhibit temporal persistence and typically occur at low abundance, though populations may exhibit an abrupt increase under certain circumstances. Our results, however, do not support any significant role of conditionally rare taxa as regards FM stability as signatures of FM consistency over time were consistently more pronounced in analyses utilizing OTU abundance compared with those based solely on OTU presence vs. absence. We conclude, therefore, that FM stability is driven by abundance invariance of relatively common OTUs and that a large proportion of OTUs that typically occur at low abundances are likely to persist for a limited period only in barn swallow FM. It could be argued that we were not able to detect any significant effect of conditionally rare taxa as these were below or at the detection threshold of our sequencing experiment. Although we cannot reject this possibility, we believe that any potential bias due to under-sequencing is unlikely to have affected the sensitivity of our analysis as high coverage index values indicate almost complete representation of FM diversity by sequencing data.

Temporal consistency effect sizes for juvenile FM sampled over several days were comparable with those observed in adults where the time-lag between samplings of the same individual was much longer, indicating a lower level of temporal FM invariance in young. This could theoretically be attributed to gradual FM succession associated with gradual changes in gut physiology and morphology and immune system during the early post-hatching period (Caviedes-Vidal and Karasov, 2001; Killpack et al., 2013). However, as our data do not provide evidence for successive changes in FM, we suggest that stochastic turnover of FM species is more rapid in juveniles. Knowledge regarding changes in FM stability in different ontogenetic stages remains relatively poor; however, our results are consistent with data from captive mice, where juveniles exhibited more rapid FM changes at the individual level compared with adults (Schloss et al., 2012). In addition, high stochasticity of microbial communities in early life stages is implied by numerous studies showing their dramatic changes during postnatal development (Schloss et al., 2012; Bäckhed et al., 2015; Mach et al., 2015).

Further evidence for increased stochasticity in the FM composition of juveniles was provided by our OTU-level analysis, which showed that only OTUs represented by a very low proportion of 16S rRNA reads, exhibited signs of temporal stability in juveniles, whereas both the number of OTUs with stable abundance over time and their relative representation in FM was much higher in the case of adults. Seventeen OTUs contributed significantly to FM stability in adults, both at the within- and between-season time-scale, and these represent potential candidates for involvement in long-term modulation of the host phenotype. Further correlative and experimental research, therefore, should focus on the relevance of these 17 OTUs as regards host traits involved in fitness pay-offs and mechanisms maintaining their temporal stability. At present, the role of individual FM species on host fitness in passerines is little understood (González-Braojos et al., 2012b; Benskin et al., 2015); nevertheless, the relevance of OTUs exhibiting consistent signs of temporal stability is indicated by numerous studies suggesting both beneficial and harmful effects in humans and other organisms. In barn swallow, several of these OTUs were Actinobacteria (genus *Dietzia, Corynebacterium* and *Tsukamurella*), i.e., they belong to a bacterial clade characterized by production of bacteriocins and other secondary metabolites involved in interactions with other microbiota members (Riley and Wertz, 2002), implying their importance for FM structure. *Acinetobacter* genus (phylum Proteobacteria) are able to degrade chitin (Askarian et al., 2012), which may be of substantial importance for the barn swallow given its dependence on a chitin-rich diet. Genus *Mycoplasma* (phylum Tenericutes) include several potentially pathogenic species for birds (Sumithra et al., 2013). These bacteria support mechanisms enabling adherence to epithelial cells, implying a tight interaction with the host’s immune system (Chu et al., 2003). Notably, the Lactobacillus OTU (corresponding to *L. reuteri* by blast search; 100% identity), known for its probiotic properties in poultry (Liu et al., 2007), exhibits a substantial level of temporal stability in barn swallow FM, as did *Streptococcus* OTU populations (corresponding to S. *thermophilus* by blast search; 100% identity), which also have a putative probiotic effect (Correa et al., 2005). Importantly, other abundant taxa of putatively environmental origin, such as Xantomonadaceae and Alphaproteobacteria, were not present in the OTU subset exhibiting signs of temporal stability, suggesting transient colonization of the gut by bacteria from the environmental pool as an important source of FM temporal variation at the within-individual level in barn-swallows. Although further research is needed to distinguish transient vs. resident members in passerine FM, a large proportion of putatively transient bacteria are probably associated with rapid passage of food through the digestive tract (Caviedes-Vidal et al., 2007; McWhorter et al., 2009), which may preclude their effective elimination or overgrowth by resident bacteria. Aside from the effect of transient bacteria, within-individual variation could theoretically be explained by a wide range of factors, including temporal changes in hormonal profiles or immune parameters (Koren et al., 2012; Org et al., 2016). Our own unpublished data suggest a tight association between cell-mediated immune response and FM composition in barn swallow, which is consistent with the latter explanation (Kreisinger et al., unpublished data). On the other hand, while there are contrasting differences in male and female hormonal profiles during the breeding season (Garamszegi et al., 2005), sex has been shown to have a negligible effect on FM composition in barn swallow (Kreisinger et al., 2015b). We therefore speculate that physiological changes modulated by variation in hormonal levels are unlikely to explain temporal variation in FM at the individual level.

### FM Similarity between Offspring and Their Parents

Strict vertical transmission of FM from parents to progeny plays an important role in host vs. host-associated microbiota co-adaptations in several arthropod taxa, including the evolution of obligatory symbiotic interactions between host-associated microbiota members and their host. This results in a tight phylogenetic co-divergence between host-associated microbiota and their host (Janson et al., 2008), and perhaps in Dobzhansky–Muller type incompatibilities due to host-associated microbiota admixture in hybrids (Brucker and Bordenstein, 2012). In vertebrates, host-associated microbiota vs. host associations mediated by trans-generational transfer are probably not so tight; nevertheless, this mechanism still plays a significant role in genome evolution in some host-associated microbiota species (Falush et al., 2003). In mammals, initial inoculation of newborn young by vaginal microbiota during the delivery has a long-term effect on their FM composition (Salminen et al., 2004), while prebiotic compounds included in breast milk facilitate proliferation of beneficial microbes in the gut (Bode, 2012). The role of such parental effects on the FM of progeny has generally been poorly explored in non-viviparous vertebrate taxa (Lucas and Heeb, 2005). Where post-natal parental care exists in non-viviparous species, however, bacteria are likely to be transferred during food provisioning or other types of physical contact between parents and offspring (Lucas and Heeb, 2005). In barn swallow, we observed significantly higher similarity in FM composition between social mothers and their offspring but not in the case of FM comparisons between offspring and social fathers. The lower effect of social fathers on FM composition in progeny can be explained by the lower contribution of male barn swallows to parental care (Smith and Montgomerie, 1992; Møller, 1994). This results in a lower rate of social contact, which has previously been shown to shape microbial communities in birds (White et al., 2010; Kreisinger et al., 2015b). Although direct FM transfer during food provisioning is the most parsimonious explanation for mother vs. offspring similarity, we cannot exclude the possibility that it is mediated by *in ovo* maternal deposition of bioactive compounds that have the potential to modulate FM (Yurkovetskiy et al., 2013). On the other hand, we believe that mother vs. offspring similarity is unlikely to be caused by vertical inheritance of genes interacting with FM as a paternal effect on offspring FM was of a much lower effect-size and did not increase after expulsion of extra-pair (i.e., non-genetic) offspring, and mitochondria and heterogametic sex chromosomes are the only parts of the genome inherited exclusively maternally, and these are unlikely to have any considerable effect on FM structure (Benson et al., 2010; McKnite et al., 2012). Finally, there is some evidence for egg (and consequently embryo) inoculation by bacterial populations in the uterus (Funkhouser and Bordenstein, 2013). How often this occurs, however, and how this mechanism contributes to FM after hatching, remains unclear.

### Conclusions

The aim of this study was to assess the strength of FM composition temporal consistency and the level of parental effect on juvenile FM composition. Both these factors are important from an ecological and evolutionary perspective as they help promote within individual and trans-generational consistency of phenotype traits linked with FM. Our data, however, revealed a limited role for these two factors.

At the whole community level, FM exhibited significant temporal consistency, both in adults and juveniles, though corresponding effect sizes were low. Nevertheless, we identified a subset of bacteria whose relative abundances exhibited pronounced levels of temporal consistency in adults, both at the within- and between-year time-scales. Consequently, these OTUs may be involved in long-term modulation of the host phenotype. This possibility, along with identification of the mechanisms underlining stability of these OTUs over time, should be the subject of further empirical evaluation.

Our data also indicate a slight maternal, but not paternal, effect on FM composition in social offspring. This pattern may be explained by direct social transfer of FM, which has been proposed as a mechanism underlining gut microbiota heritability. Our data, however, are not fully consistent with this idea. The observed effect size of mother vs. offspring similarity was low and did not increase with offspring age. Consequently, pronounced differences between juvenile and adult microbiota are unlikely to be compensated for by a maternal effect during the nestling stage. Thus, the switch between juvenile-specific and adult-specific FM likely takes place after nest abandonment, when any parental effect is presumably to be limited. In addition, juvenile FM composition was highly variable during the nestling stage and, consequently, maternal effect on offspring FM is likely to persist for a limited period only.

## Data Accessibility

Raw FASTQ files: http://www.ebi.ac.uk/ena/data/view/PRJEB14586

## Author Contributions

Design of the study: JK and TA. Field sampling: TA, RM, AP, MA, OT, and JK. Laboratory analysis: LK, J-FM, and RM. Data analysis: JK. Financial funding: JK, TA, and LK. Manuscript drafting: JK, LK, and TA. All authors provided helpful comments and recommendations and approved the final version of the manuscript.

## Conflict of Interest Statement

The authors declare that the research was conducted in the absence of any commercial or financial relationships that could be construed as a potential conflict of interest.

## Footnotes

1 http://sourceforge.net/projects/microbiomeutil/files

## Supplementary Material

**TABLE S1 | Detailed listing of barn swallow samples.**

**TABLE S2 | Taxonomic composition of barn swallow FM and its variation between adults and juveniles.**

**DATA SHEET 1 | Supplementary methods and analysis.**
