# Supplementary material for: Temporal Stability and the Effect of Transgenerational Transfer on Fecal Microbiota Structure in a Long Distance Migratory Bird
Source: Front Microbiol. 2017 Feb 1;8:50. doi: 10.3389/fmicb.2017.00050 (PMC5292904; doi:10.3389/fmicb.2017.00050)
Supplement: Supplementary file 3 [file Data_Sheet_1.PDF]

Supplementary Material

Temporal stability and the effect of transgenerational transfer on faecal microbiota structure in a long distance migratory bird

Jakub Kreisinger*, Lucie Kropáčková, Adéla Petrželková, Marie Adámková, Oldřich Tomášek, Jean-François Martin, Romana Michalková, Tomáš Albrecht

***Correspondence:** Jakub Kreisinger; jakubkreisinger@seznam.cz

1. Supplementary method

1.1. Details on permutation procedures

The following section provides details on the permutation procedures that were used to test temporal stability of gut microbiota (FM) and FM divergence among nests, breeding colonies and breeding seasons in juveniles and adults. A similar description is provided for analysis of FM similarity among social parents and their offspring.

For each test performed in the main text, we briefly describe the underlining hypothesis and list the types of dissimilarity that were excluded before calculation and the blocking variables (i.e. strata) that were setup as permutation constraints in order to avoid biased inference.

1.1.1. Analysis of FM divergence in adults

[A] Among vs. within year dissimilarities:

Hypothesis: FM composition differs among breeding seasons after statistical control for FM divergence among breeding colonies.
Excluded dissimilarities: Dissimilarities among samples from the same individual and dissimilarities among samples from different colonies.
Permutation constraints: Colony.
Number of pair-wise dissimilarities: Within-year comparisons = 10660, among-year comparisons = 9788.

[B] Among vs. within colony dissimilarities:

Hypothesis: FM composition differs among breeding colonies after statistical control for FM divergence among breeding seasons.
Excluded dissimilarities: Dissimilarities among samples from the same individual and dissimilarities among samples from different years.
Permutation constraints: Breeding season.
Number of pair-wise dissimilarities: Within-colony comparisons = 5429, among-colony comparisons = 10660.

[C] Among vs. within individuals (different season)

Hypothesis: There is higher similarity among samples from the same individual than among samples from different individuals collected during different breeding seasons.
Excluded dissimilarities: Dissimilarities among samples from the same year.
Permutation constraints: Breeding colony.
Number of pair-wise dissimilarities: Within-individual comparisons = 71, among-individual comparisons = 4829.

[D] Among vs. within individuals (same season)

Hypothesis: There is higher similarity among samples from the same individual than from different individuals collected during the same breeding season.
Excluded dissimilarities: Dissimilarities among samples from different years and dissimilarities among different colonies.
Permutation constraints: Breeding season and breeding colony.

Number of pair-wise dissimilarities: Within-individual comparisons = 99, among-individual comparisons = 5429.

1.1.2. Analysis of adult FM divergence in juveniles

[A] Among vs. within colony dissimilarities:

Hypothesis: There is a difference in FM composition among breeding colonies.
Excluded dissimilarities: Dissimilarities among samples from the same nest and dissimilarities among samples of different age-classes.
Permutation constraints: Age-class.
Number of pair-wise dissimilarities: Within-colony comparisons = 6327, among-colony comparisons = 12979.

[B] Among vs. within nest dissimilarities:

Hypothesis: There is higher similarity among samples from the same nest than in different nests after control for variation among different age-classes and breeding colonies.
Excluded dissimilarities: Dissimilarities among samples from the same individual, among samples from different breeding colonies and from different age categories.
Permutation constraints: Breeding colony and age-class.
Number of pair-wise dissimilarities: Within-nest comparisons = 213, among nest comparisons = 2436.

[C] Among vs. within individuals

Hypothesis: There is higher similarity among samples from the same individual than in different individuals from the same nest.
Excluded dissimilarities: Dissimilarities among samples from different nests and dissimilarities among juveniles of the same age.
Permutation constraints: Nest identity.
Number of pair-wise dissimilarities: Within-individual comparisons = 123, among-individual comparisons = 385.

1.1.3. Analysis of FM similarity between social parents and their offspring.

The following procedure was applied to account for our dataset including multiple samples from the same social parent and their offspring:

As a first step, we randomly selected samples corresponding to unique social father vs. offspring pairs (n = 37 pairs) and social mother vs. offspring pairs (n = 31 pairs). We then calculated community dissimilarity within this sample subset. Juvenile vs. juvenile and adult vs. adult dissimilarities were then excluded and dissimilarities observed among offspring vs. parental pairs were compared with dissimilarities among juveniles and unrelated males and juveniles and females. A null distribution of dissimilarities was generated by random permutation, as described above and in the main text, and this was subsequently used for testing significance.

This process was repeated 20-times, the results presented in the main text corresponding to an average of these 20 repetitions.

2. Supplementary results

2.1. Differentiation within predicted metagenomes for adults and young

The PICTUSt algorithm was employed to predict metagenome composition of barn swallow FM. Description of downstream statistical analysis of PICRUSt predictions are provided in the main text. In brief, divergence in the composition of predicted metagenomes between adults and juveniles was visualised using principal coordinate analysis (PCoA; Figure S1). DeSeq2 pipeline was then used to identify KEGG (i.e. Kyoto Encyclopedia of Genes and Genomes) categories whose predicted abundances differ between adults and juveniles (Figure S2).

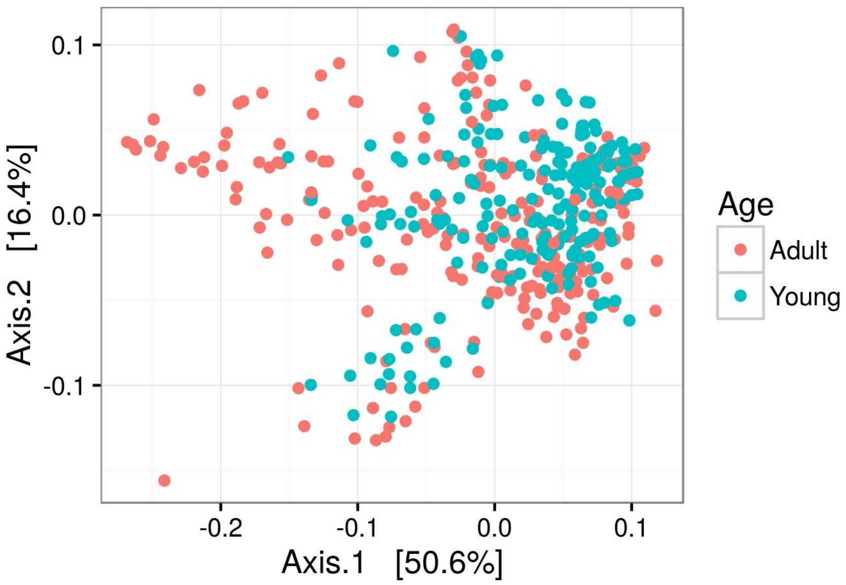

Figure S1: Principal coordinate analysis *for Bray-Curtis dissimilarities among predicted metagenomes of juvenile (blue) and adult (red) barn swallows. The proportion of variation associated with individual axes is indicated in the plot.*

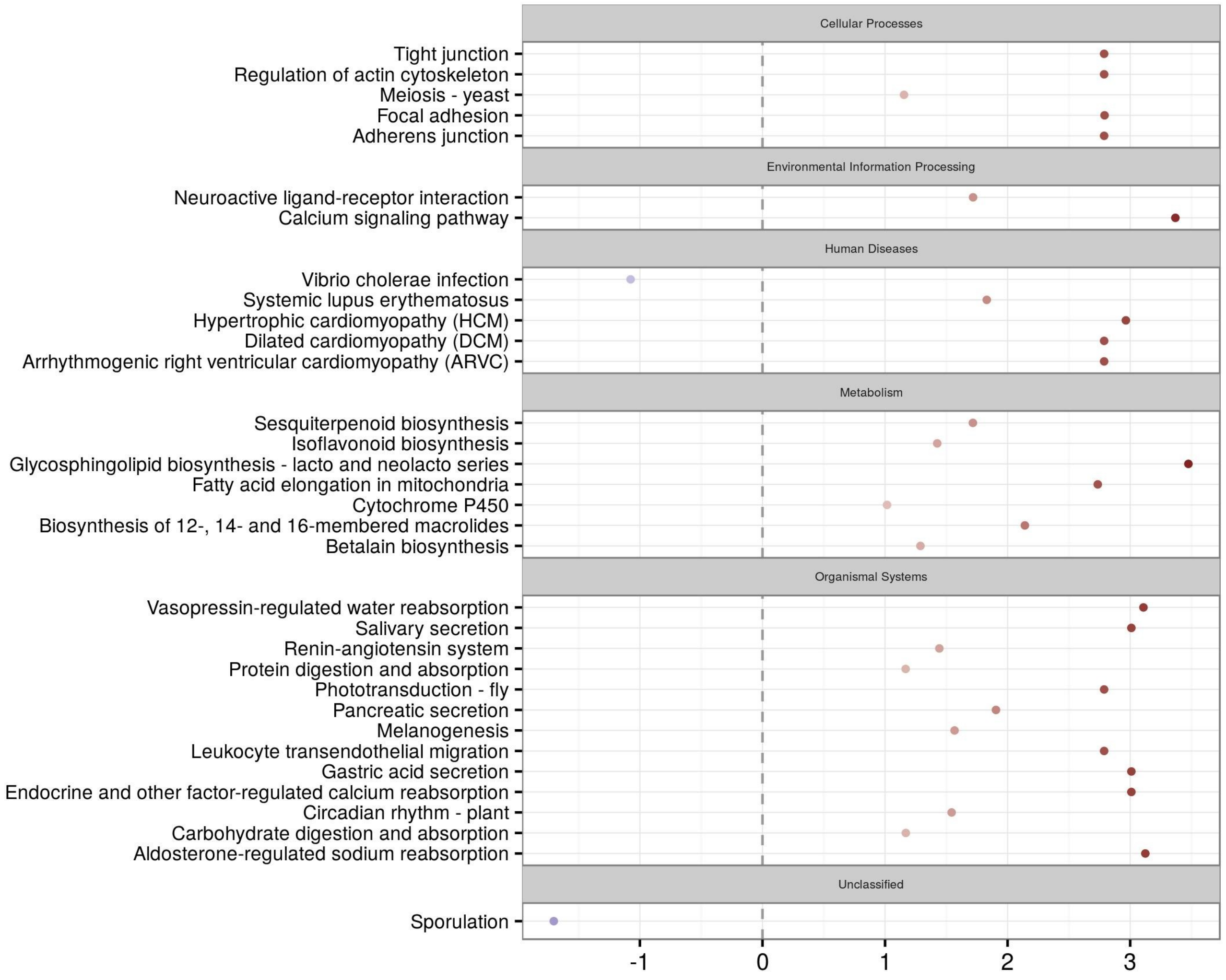

Figure S2: Differential abundance analysis for abundance of predicted KEGGs in juvenile vs. adults barn swallows. *Position on the horizontal axis shows* log_2 *fold changes calculated using DESeq2 pipeline. Negative (blue) and positive (red) values indicate higher abundance of a given KEGG category in juveniles and adults, respectively. Only KEGG categories where the absolute value of the* log_2 *fold change was > 1 are shown.*

2.2. Extended analysis of patterns in operational taxonomic unit (OTU) co-occurrence

Generalised linear mixed effect models with Poisson errors were used to identify taxonomic groups that were significantly more or less likely to be involved in co-occurrence or co-avoidance associations compared to the whole FM baseline. See the main text for a more detailed description of the corresponding procedures. Below, we provide a graphical representation of random effect estimates inducing approximate 95% confidence intervals for bacterial classes that were represented by at least 10 OTUs.

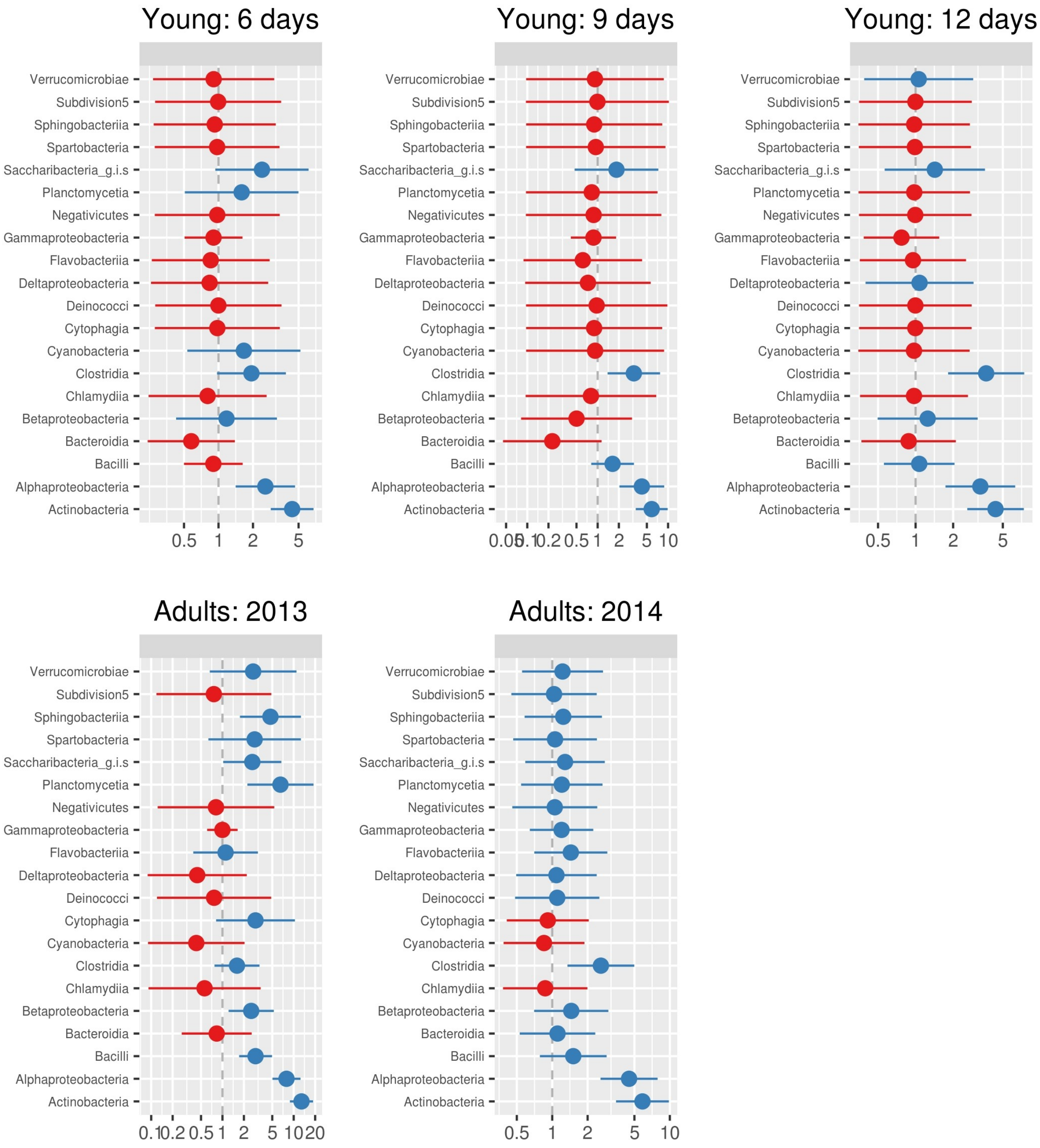

Figure S3: *Deviation in the number of significant co-occurrence and co-avoidance interactions per OTU from the whole FM baseline for individual bacterial classes and age categories. Presented are estimates of random effects ± 95% confidence intervals derived based on generalised mixed effect models.*

2.3. Extended analysis of FM temporal invariance and its divergence among nests, breeding colonies and years

Permutation-based tests were applied in order to assess whether dissimilarity among samples corresponding to the same adult or young individual, same nest, breeding colony or breeding season was lower than among samples that did not match these categories. Details on the permutation procedures applied are provided above and in the main text. The analysis was run on four community-dissimilarity types in the case of OTU data and on Bray-Curtis dissimilarities in the case of metagenomic predictions. Here we present graphical outputs of these analyses for three dissimilarity types calculated based on OTU data that were not presented in the main text (Figures S4 and S5) as well as for metagenomic predictions (Figures S6 and S7). Results on parent vs. offspring similarity in FM compositions are in Figures S8 and S9.

In addition, the same permutation analyses were performed using Bray-Curtis dissimilarities calculated for individual OTUs in order to identify those whose abundances exhibited significant levels of stability over time (i.e. higher among that within individual divergence, as detailed in the main text). Based on these analyses, only those OTUs that exhibited significantly lower divergence at the within-individual level were retained in the OTU tables. A graphical representation of these results is provided in Figure S10.

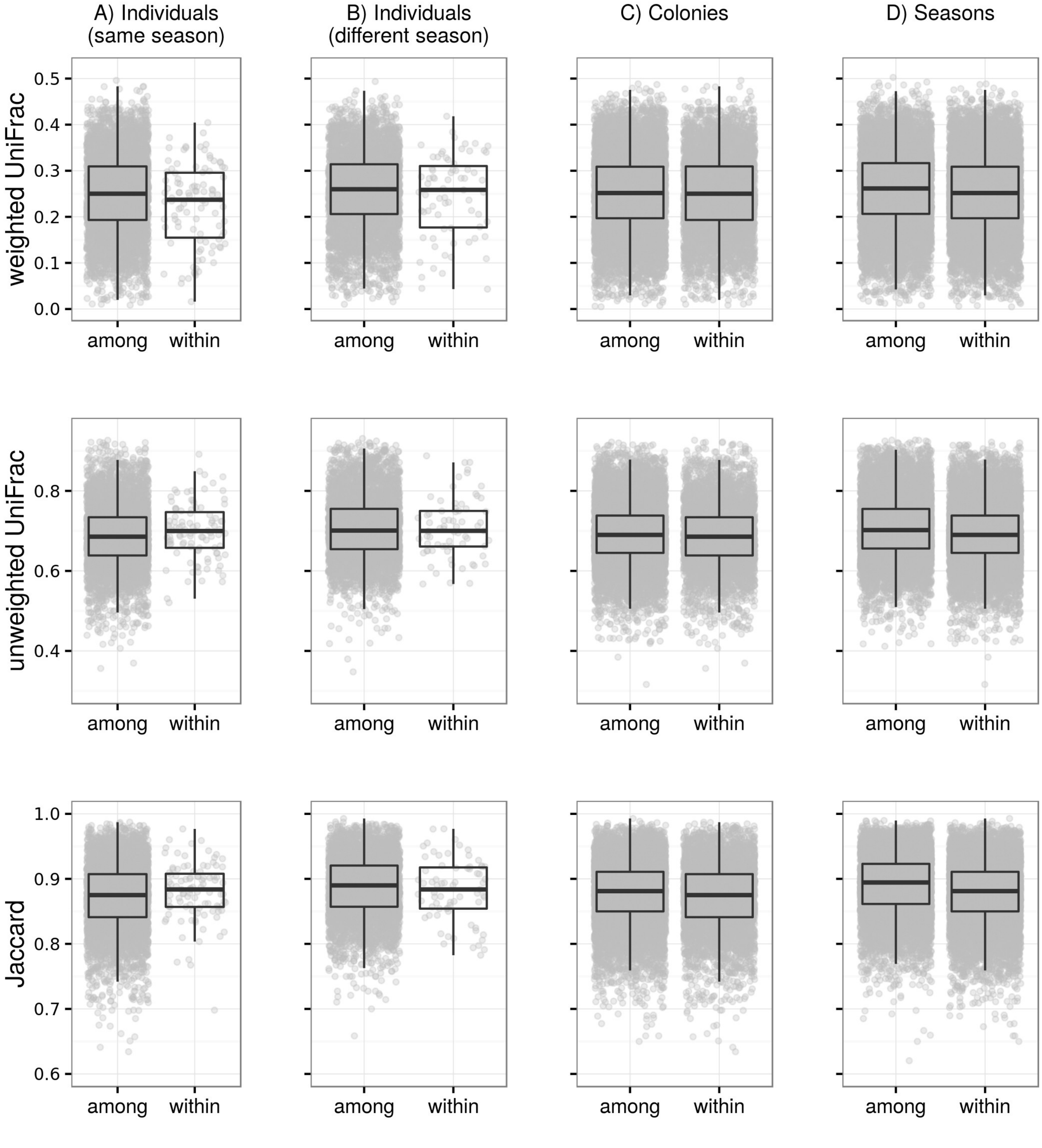

Figure S4: FM differentiation in adult barn swallows. *Box plots for dissimilarity among FM samples from identical vs. non-identical individuals sampled A) during the same and B) during different breeding seasons, C) dissimilarities among FM samples from non-identical individuals sampled during the same breeding season in the same colony vs. different colony, and E) dissimilarities among FM samples from non-identical individuals sampled in the same breeding colony during the same breeding season vs. different breeding season. These analyses were performed using four types of ecological dissimilarity. Here we provide results for weighted and unweighted UniFrac and Jaccard dissimilarities.*

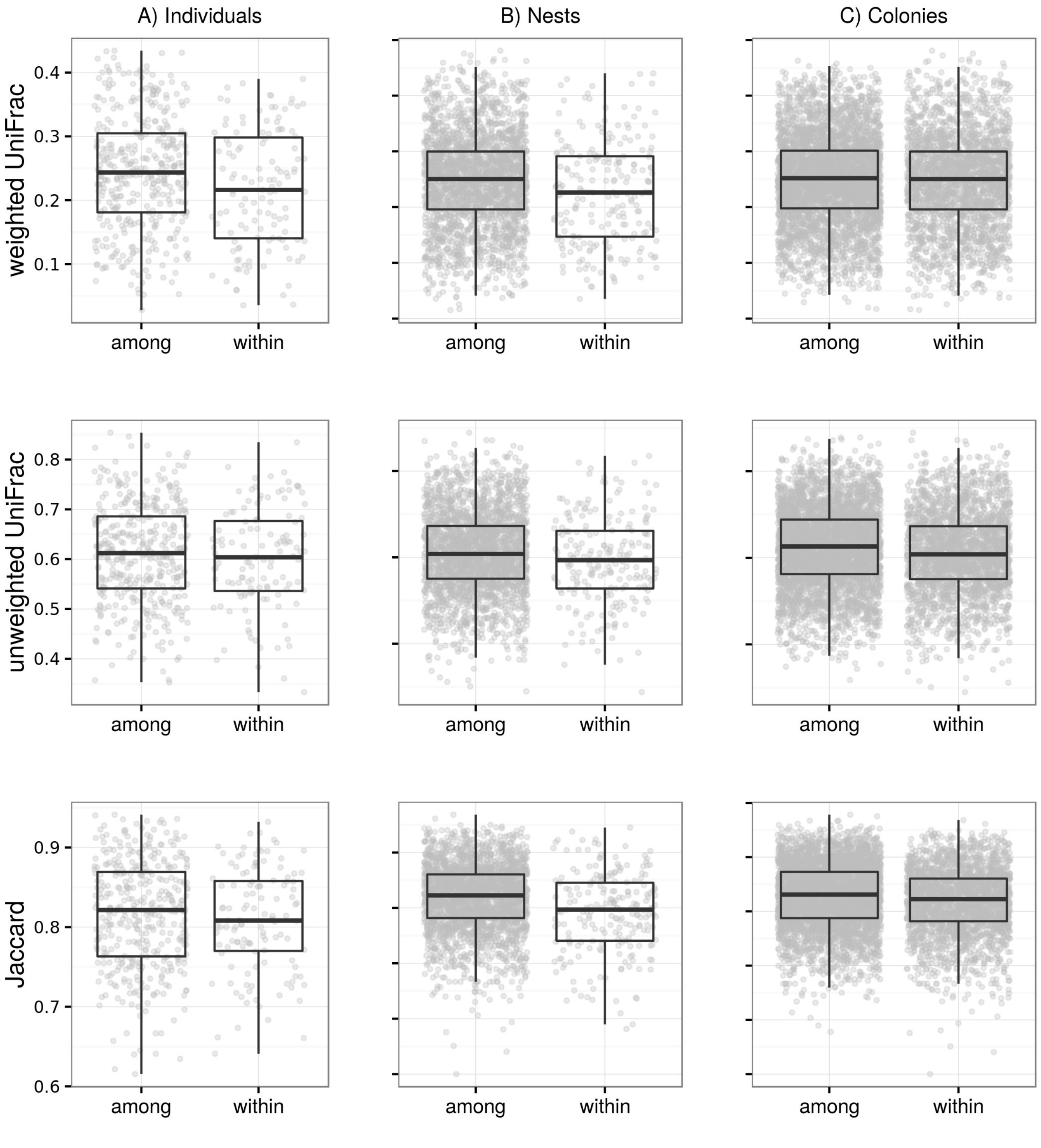

Figure S5: FM differentiation in juvenile barn swallows. *Box plots for dissimilarity among FM samples from A) identical vs. non-identical individuals from the same nest corresponding to different age-classes, B) non-identical individuals from identical vs. non-identical nests placed in the same breeding colony and corresponding to same age-class, and C) individuals from non-identical nests that were placed in the same vs. different breeding colony. Here we provide results for weighted and unweighted UniFrac and Jaccard dissimilarities.*

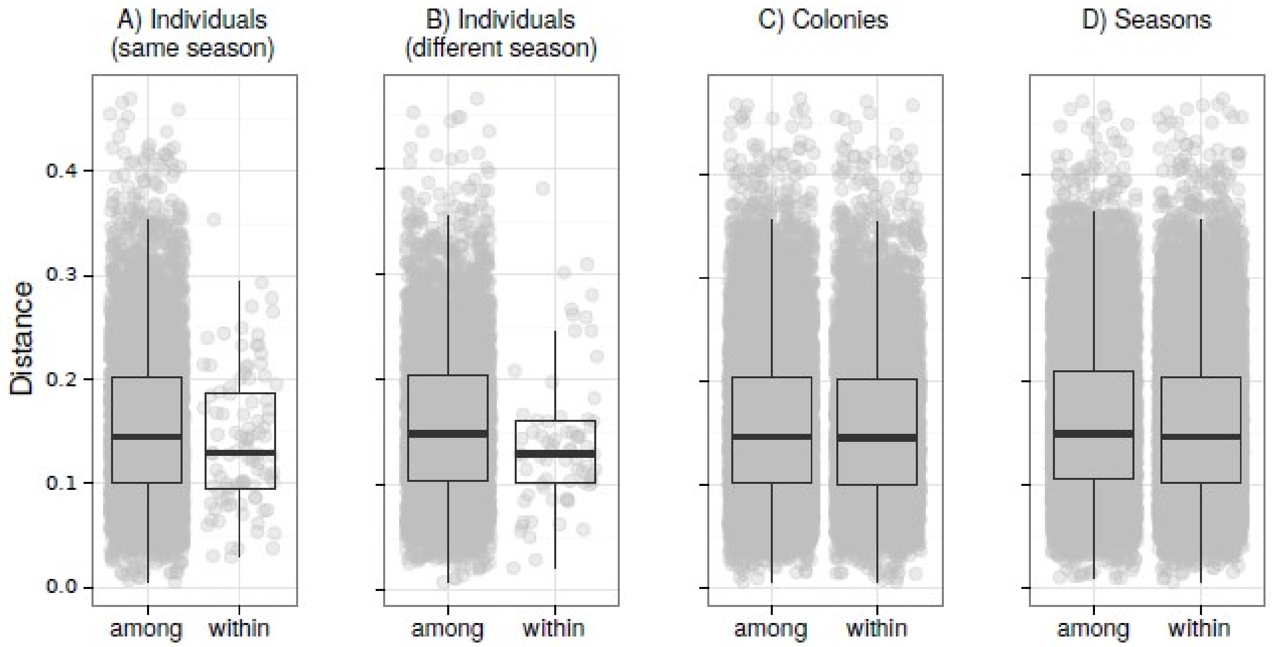

Figure S6: Differentiation of predicted metagenome composition in adult barn swallows. *Box plots for Bray-Curtis dissimilarity among predicted metagenomes for individual samples from identical vs. non-identical individuals sampled during A) the same breeding season and B) during different breeding seasons, C) dissimilarities among FM samples from non-identical individuals sampled during the same breeding season in the same vs. different colony, and E) dissimilarities among FM samples from non-identical individuals sampled in the same breeding colony during the same vs. different breeding season.*

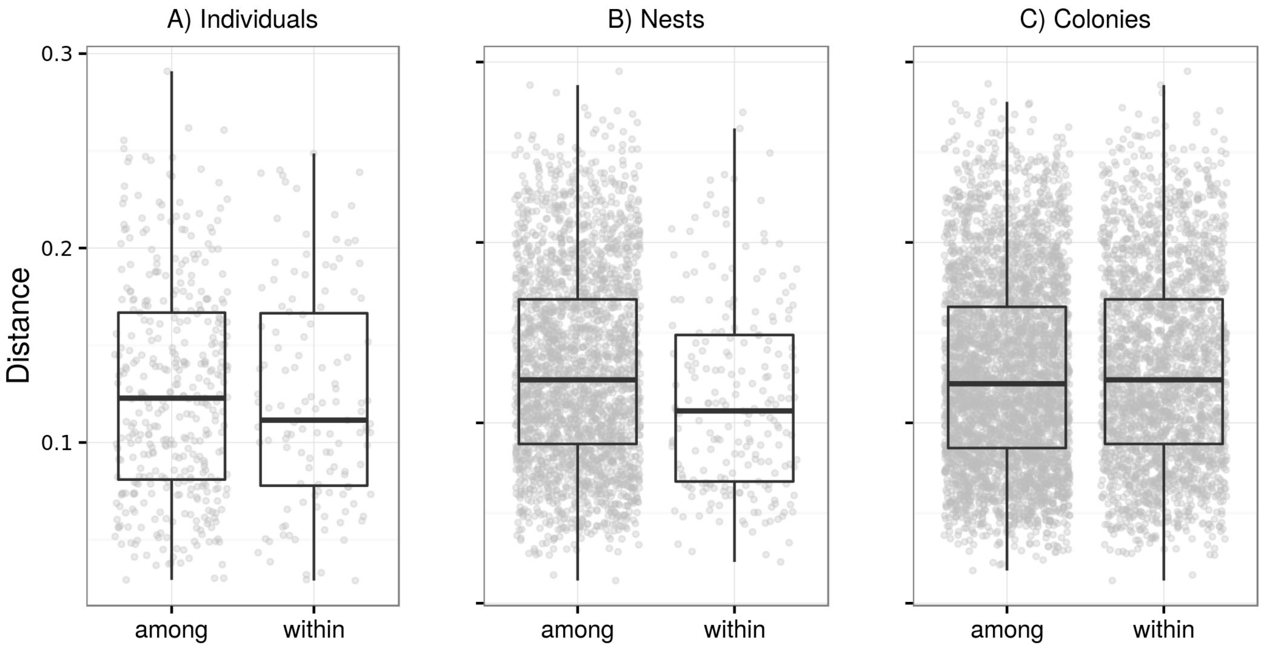

Figure S7: Differentiation of predicted metagenome composition in juvenile barn swallows. *Box plots for Bray-Curtis dissimilarity among predicted metagenomes for A) identical vs. non-identical individuals from the same nest corresponding to different age-classes, B) non-identical individuals from identical vs. non-identical nests placed in the same breeding colony corresponding to the same age-class, and C) individuals from non-identical nests that were placed in the same vs. different breeding colony.*

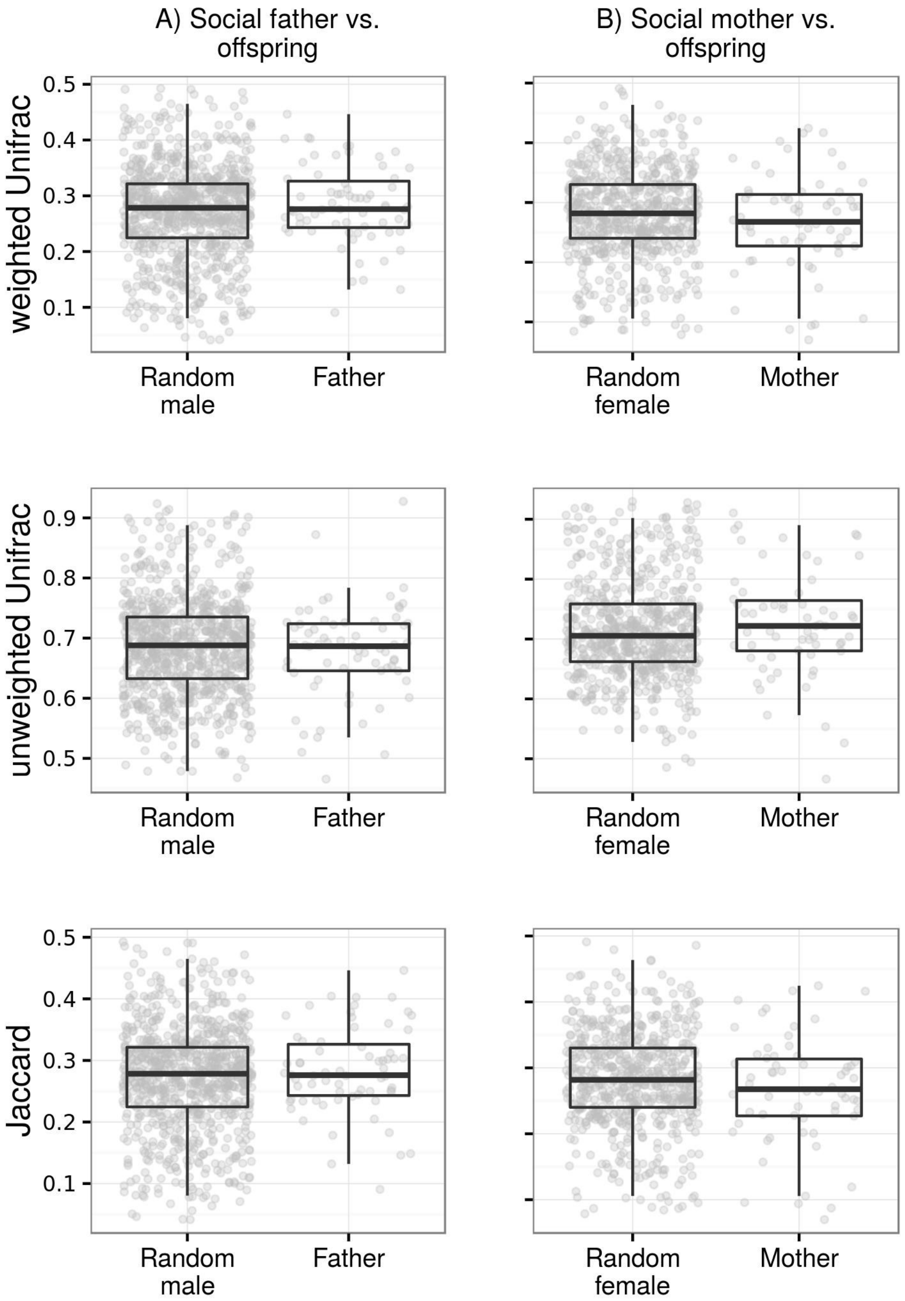

Figure S8: FM similarity between young barn swallows and their social parents. *Box plots for FM dissimilarity among juveniles and A) their social fathers vs. non-social adult males and B) social mothers vs. non-social adult females. Here we provide results for weighted and unweighted UniFrac and Jaccard dissimilarities.*

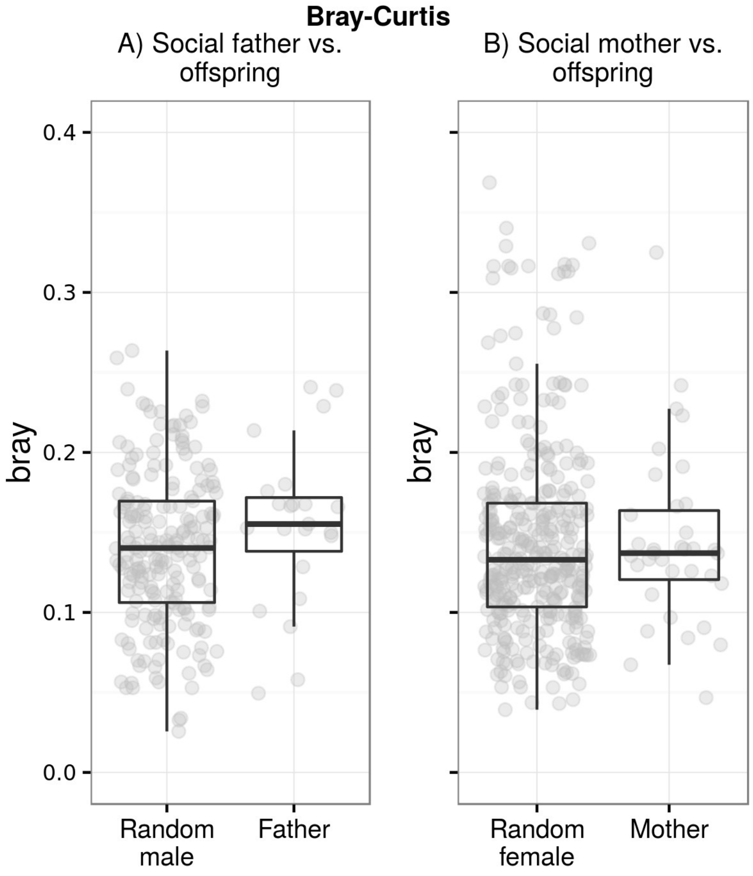

Figure S9: Similarity of predicted metagenomes between young barn swallows and their social parents. *Box plots for Bray-Curtis dissimilarity in predicted metagenomes among juveniles and A) their social fathers vs. non-social adult males and B) social mothers vs. non-social adult females.*

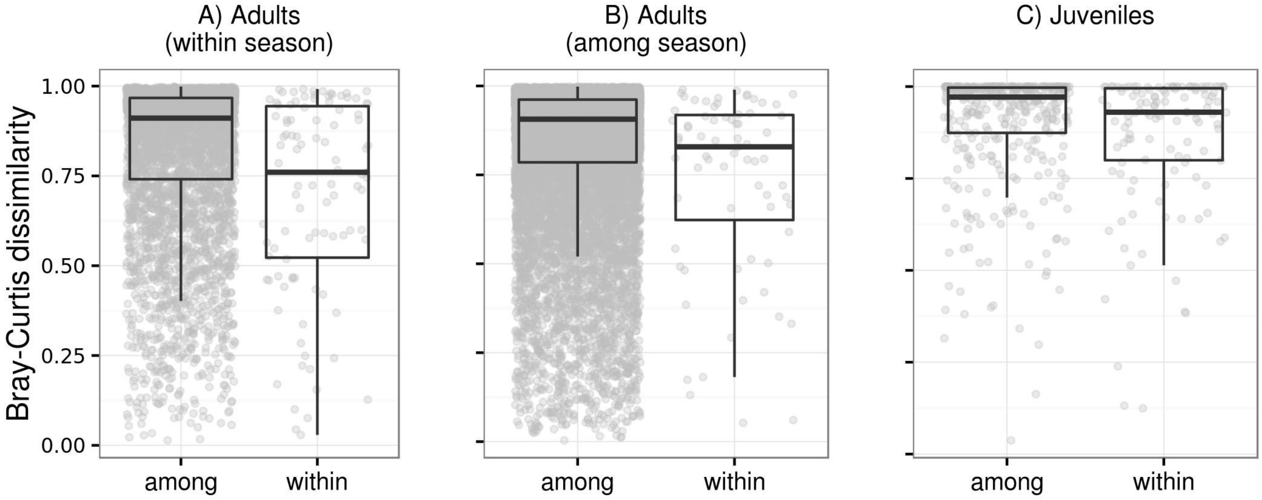

Figure S10: FM divergence within vs. among barn swallow individuals based on OTUs exhibiting temporal stability. *Box plots for Bray-Curtis dissimilarity among FM samples from identical vs. non-identical A) adult individuals sampled during the same breeding season, B) adult individuals sampled during different breeding seasons, and C) juveniles from the same nest. Dissimilarities were calculated based on a subset of the original OTU table that included only those OTUs whose abundances exhibited signs of temporal consistency (i.e. significantly lower divergence within than among individuals).*
